# Conserved γδ T cell selection by BTNL proteins limits progression of human inflammatory bowel disease

**DOI:** 10.1126/science.adh0301

**Published:** 2023-09-15

**Authors:** Robin J Dart, Iva Zlatareva, Pierre Vantourout, Efstathios Theodoridis, Ariella Amar, Shichina Kannambath, Philip East, Timothy Recaldin, John C Mansfield, Christopher A Lamb, Miles Parkes, Peter M Irving, Natalie J Prescott, Adrian C Hayday

**Affiliations:** 1 Peter Gorer Dept of Immunobiology, King’s College London at Guy’s Hospital Campus, London, United Kingdom; 2 Immunosurveillance Laboratory, The Francis Crick Institute, London, UK; 3 Department of Gastroenterology, Guy’s and St Thomas’ Foundation Trust, London, UK; 4 Department of Medical and Molecular Genetics, King’s College London, London, UK; 5 NIHR BRC Genomics Centre, King’s College London, London, UK; 6 Bioinformatics and Biostatistics, The Francis Crick Institute, London, UK; 7 GammaDelta Therapeutics Ltd., London, UK; 8 Translational & Clinical Research Institute, Newcastle University, Newcastle upon Tyne, UK; 9 Department of Gastroenterology, Newcastle upon Tyne Hospitals NHS Foundation Trust, Royal Victoria Infirmary, Newcastle upon Tyne, UK; 10 Department of Medicine, Addenbrooke’s Hospital, University of Cambridge, Cambridge, UK

## Abstract

Murine intraepithelial γδ T cells include unique, tissue-protective cells selected by epithelial Butyrophilin-like (BTNL) heteromers. Interrogating whether this biology is conserved in humans, we characterized the colonic γδ T cell compartment, identifying a diverse repertoire that includes a phenotypically distinct subset co-expressing T cell receptor Vγ4 and the epithelium-binding integrin CD103. This subset was disproportionately diminished and dysregulated in inflammatory bowel disease (IBD), whereas on-treatment CD103^+^γδ T cell restoration was associated with sustained IBD remission. Moreover, CD103^+^Vγ4^+^cell dysregulation and loss were also displayed by humans with germline BTNL3/BTNL8 hypomorphism, which we identified as a risk factor for penetrating Crohn’s disease. Thus, BTNL-dependent selection and/or maintenance of unique, tissue-intrinsic γδ T cells appears as an evolutionarily conserved axis limiting progression of a complex, multifactorial, tissue-damaging disease of increasing global incidence.

Central and peripheral repertoire selection shape adaptive T cell immunity, purging repertoires of autoreactive T cells while enriching for those suited to recognising diverse pathogens. Thus, polymorphisms in major histocompatibility complex (MHC proteins) that present antigen to T cells can perturb repertoire selection and show causal associations with autoimmune and auto-inflammatory diseases (1).

However, many T cells, particularly γδ T cells, are not MHC restricted (2). In mice, for example, intraepithelial γδ T cell compartments of limited T cell receptor (TCR) diversity are selected for and/or maintained by TCR-dependent interactions with organ-specific Butyrophilin-like (BTNL) proteins expressed by epithelial cells (3–5). The consequent γδ T cell repertoires appear to play major roles in maintaining and restoring tissue homeostasis, with γδ T cell deficient mice showing increased susceptibility to hapten-elicited atopic dermatitis and to chemically induced colitis (6–10). Moreover, a recently reported anti-inflammatory role for mouse intestinal γδ T cells was manifest in their protection of Paneth cells dysregulated by an IBD-associated polymorphism in *ATG16L1* (11). Hence, there are potentially profound implications of the identification in humans of colonic, TCRγδ^+^ intraepithelial lymphocytes (IEL), a subset of which expresses Vγ4 chains that can directly bind heteromers of BTNL3+BTNL8 displayed by colonic epithelium (12, 13).

Nonetheless, to our knowledge, there is to date no evidence that BTNL3+BTNL8 acts as a selection/maintenance factor for human intestinal IEL in a manner akin to murine IEL selection; no clarity as to how BTNL-selected cells might differ from other gut T cells; and no resolution of whether or not they are even the major gut γδ T cell subset. Likewise, despite reports that BTNL3 and BTNL8 expression can be substantially reduced in IBD tissue samples, as well as in celiac disease (14, 15), no evidence exists linking the BTNL3+BTNL8 - Vγ4 T cell axis to the regulation of IBD and/or its treatment.

## Results

### Human gut γδ T cells are diverse, with distinct phenotypes

We first sought to elucidate the composition of the human colonic γδ T cell compartment, so that we could more fully understand the nature of dysregulation in IBD. Thus, we established a clinical workflow permitting the analysis of intraepithelial γδ T cells from colonic endoscopic biopsies from 173 adults, among whom the majority of immunological data was obtained from a core cohort of: [a] non-IBD controls (“Ctrl”) (n=34); [b] uninflamed gut from patients with confirmed IBD (“IBD”) (n=42); [c] inflamed lesions from patients with IBD (“IBDI”) (n=27); and from 7 individuals, from whom paired inflamed and uninflamed tissue was obtained at the time of endoscopy. Comprehensive donor information can be found in table S1, through which individual donors can be linked to each data-set presented.

First our samples confirmed that the colon is enriched in Vγ1^+^ cells relative to Vγ2^+^ cells that dominate the peripheral blood (2), as depicted by an illustrative flow cytometry analysis (fig. SIA) and confirmed by deep sequencing of productively rearranged TCR VDJ gene segment transcripts, among which *TRDV1* (encoding Vγ1) accounted for 60%-80% of reads (fig. SIB). By flow cytometry, most gut Vγ2^neg^ cells reacted to an antibody detecting Vγ2, Vγ3 or Vγ4 chains, and the vast majority were usually Vγ1^+^ (fig. S1C); hence colonic Vγ2^neg^ γδ T cells that are examined in several experiments below *de facto* comprise primarily Vγ2/3/4^+^Vγ1^+^ T cells.

**Figure 1:**
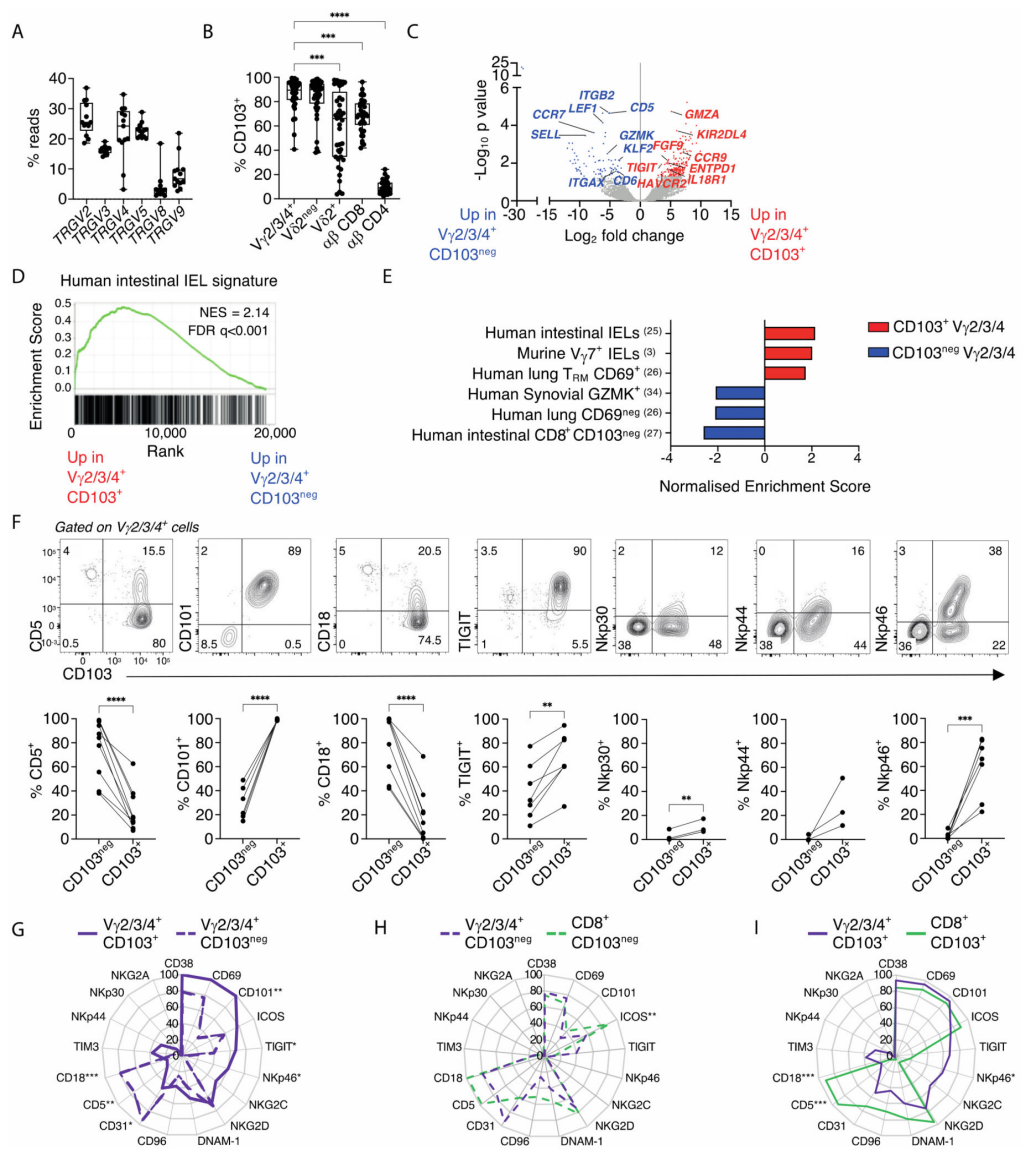
Human gut γδ T cells are diverse and predominantly intraepithelial cells with distinct phenotypes. **(A).** *TRGV* gene usage in control colonic biopsies measured by mRNA TCR deep sequencing (n=13). **(B)** Flow cytometric analysis of CD103 expression on the indicated γδ and αβ T cell subsets. (n=38-43). Median, IQR and min-max range shown. Symbols represent individual participants. Kruskal-Wallis test with Dunn’s correction against + Vγ2/3/4^+^ cells was used for analysis, ***p<0.001; ****p<0.0001. **(C).** Volcano plot of + neg + differentially expressed genes between CD103^+^ and CD103^neg^ Vγ2/3/4^+^ bulk sorted cells + from control donors (n=4, C36, C37, C39, C88). Red – significantly upregulated in CD103^+^; blue – significantly upregulated in CD103^neg^; grey – non-significant. Corrected for multiple comparisons (FDR<0.05). **(D).** Gene Set Enrichment Analysis (GSEA) of the human + + CD103 Vγ2/3/4 signature from C. in a published human natural intraepithelial lymphocyte (IEL) signature dataset (25). NES – normalized enrichment score. **(E).** Summary graph of GSEA for CD103^+^ Vγ2/3/4 (red) and CD103^neg^ Vγ2/3/4^+^ (blue) gene signatures in published datasets, (FDR<0.01). **(F).** Flow cytometric analysis of the expression of the indicated + neg+ markers (y axis) on CD103^+^ and CD103^neg^ Vγ2/3/4^+^ cells. Representative flow plots (top) and summary data (bottom) are shown (n=3 -9). Connected symbols represent individual participants. Paired *t*-tests, **p<0.01; ***p<0.001; ****p<0.0001 **(G-I).** Radar plot of the + + + neg cell surface phenotype of (G) Vγ2/3/4^+^ CD103^+^ versus Vγ2/3/4^+^ CD103^neg^ T cells; (H) Vγ2/3/4^+^ CD103^neg^ versus CD8^+^ CD103^neg^ αβ T cells; (I) Vγ2/3/4^+^ CD103 versus^+^ versus CD8^+^ CD103^+^ αβ T cells. (n=3-9 individuals per group) Plotted values represent mean of included participants. Multiple paired *t*-tests with Bonferroni-Dunn correction, *p<0.05; **p<0.01; ***p<0.001; ****p<0.0001.+

Given that BTNL-selected Vγ7 cells dominate the murine gut γδ IEL compartment (3), the reactivity of gut T cells to anti-Vγ2/3/4 might have reflected a dominance of cells expressing TCRVγ4 that mediates responsiveness to BTNL3+BTNL8 (13). However, deep sequencing revealed that *TRGV4* reads often accounted for only ~20%-35% of reads, with comparable representation of productively rearranged *TRGV2* and *TRGV5* gene segments (Fig. 1A). Moreover, high donor-to-donor variation existed for the frequencies of *TRGV4* and *TRGV2* gene rearrangements, that were inversely correlated, as reflected by paired samples (fig. S1D). There was also somewhat variable representation of *TRGV9*, the product of which (Vγ9) often, albeit not exclusively, pairs with Vδ2. However, unlike *TRGV2*, the expression levels of this and other *TRGV* genes did not correlate inversely with *TRGV4*. To conclude, whereas the dominant gut γδ T cell type expressed Vδ1, transcripts encoding BTNL-reactive Vδ4 were commonly not the most highly expressed of the Vγ chain mRNAs.

We next noted that Vγ2^+^, Vγ3^+^ and Vγ4^+^ cells that collectively accounted for over 75% of the gut V52^neg^ γδ T cell compartment (median 85.10% [95% CI 62.5-90.9; n=14]) (fig. S1C) could be segregated according to whether they expressed integrin αEβ7 (a.k.a. CD103), which was true for most cells (median 89.5% [95% CI 83.5-93.8; n=43]) (Fig. 1B), or did not express it. CD103 is typically associated with IEL and tissue-resident memory (T_RM_) CD8^+^ αβ T cells (16), with its ligand, E-cadherin, expressed by differentiated epithelial cells (17). To assess the potential significance of CD103 expression by Vγ2/3/4 lymphocytes, we compared transcriptomes of paired CD103^+^ and CD103^neg^Vγ2/3/4^+^ T cell preparations from 4 control donors (Fig. 1C; table S2). The most differentially and significantly over-expressed genes in CD103^+^Vγ2/3/4^+^ cells across all donors included those encoding: *TIGIT*, a negative regulator upregulated by BTNL-mediated selection of mouse TCRαβ^+^ IEL (3); *KIR2DL4*, an activating NK cell receptor (18); *CCR9*, associated with homing to gut epithelium (19); *GZMA*, associated with cytolytic potential (20); *IL18R*, encoding a receptor for which the ligand (IL18) promotes IFNγ production (21); *ENTPD1* (CD39), that suppresses inflammation by hydrolysing ATP (22); and *HAVCR2* (TIM3), associated with chronic TCR cell stimulation (23) (Fig. 1C). Additionally, at low expression levels, CD103^+^ γδ cells differentially expressed *FGF9*, a wound-healing factor produced by murine dermal γδ T cells (24). These specific genes combined with others to compose significant overall similarities of CD103^+^Vγ2/3/4^+^ cells to human intestinal intraepithelial T cells (25) (Fig 1D, E); to mouse Vγ7^+^ IEL (3); and to human, CD69^+^CD8^+^lung T_RM_ cells (26) (that are primarily intraepithelial) (Fig. 1E), as assessed from publicly available datasets. Conversely, overlap was less with total intestinal TCRαβ^+^CD8^+^CD103^+^ T_RM_ cells (27) (fig. S1E), considered further below.

CD103^neg^Vγ2/3/4^+^ T cells, by contrast, over-expressed genes commonly associated with circulating T cells (Fig. 1C; table S2), specifically: *SELL* (CD62L) and *CCR7* (28); integrins *ITGB2* (CD18) and *ITGAX1* (CD11c) that mediate heterotypic cell-cell interactions (29); *CD5* and *CD6*, encoding proteins associated with cell signalling thresholds (30, 31); *KLF2* and *LEF1*, commonly co-expressed in naïve, memory and/or quiescent CD8^+^ T cells (32) with *LEF1* implicated in suppressing IL17 production by mouse γδ T cells (33); and *GZMK*, associated with tissue-infiltrating human CD8^+^αβ T cells of reduced cytolytic but high cytokine-producing potential (34) (Fig. 1C). Indeed, there was an overall significant similarity to tissue-infiltrating human synovial CD8^+^αβ T cells (34); to human lung T cells lacking CD69 (26); and to human intestinal CD103^neg^CD8^+^ T cells (27) (Fig. 1E).

In validating transcriptomic differences by flow cytometry, we noted as expected that neither CD103^+^ nor CD103^neg^ cells were homogeneous, with approximately two-thirds of CD103^+^ cells expressing TIGIT and/or TIM3, while CD103^neg^ cells were mosaic for TIGIT and also for the costimulatory receptor ICOS (Fig. 1F; fig. S1F). Despite this, there was a consensus CD103^+^ cell phenotype, that was CD69^+^CD101^+^ICOS^+^CD18^neg^, whereas most CD103^neg^ cells were CD101^neg^CD5^+^CD18^+^CD31^+^TIM3^neg^ (Fig. 1F, G; fig. S1F). In conclusion, CD103 segregated Vγ2/3/4^+^ T cells into qualitatively distinct subsets (Fig. 1G). Moreover, whereas CD103^neg^Vγ2/3/4^+^ cells closely resembled human intestinal CD103^neg^TCRαβ^+^CD8^+^ cells (Fig. 1E, H), CD103^+^Vγ2/3/4^+^ cells appeared unique, being clearly distinguishable from human tissue-resident CD103^+^TCRαβ^+^CD8^+^ cells, e.g., by low expression of CD5 and CD18 (Fig. 1I). Also of note, CD103^+^Vγ2/3/4^+^ cells often expressed several NK receptors, including NKp44, NKG2C, and NKp46 that were mostly expressed by neither CD103^neg^Vγ2/3/4^+^ cells nor CD103^+^TCRαβ^+^CD8^+^ cells (Fig. 1F, I; fig. S1F). In contrast, all subsets expressed comparably high levels of NKG2D (fig. S1F), that can mediate innate responsiveness and co-stimulate TCR-mediated adaptive responses (35).

### Selective responsiveness of human gut γδ T cells

**Figure 2:**
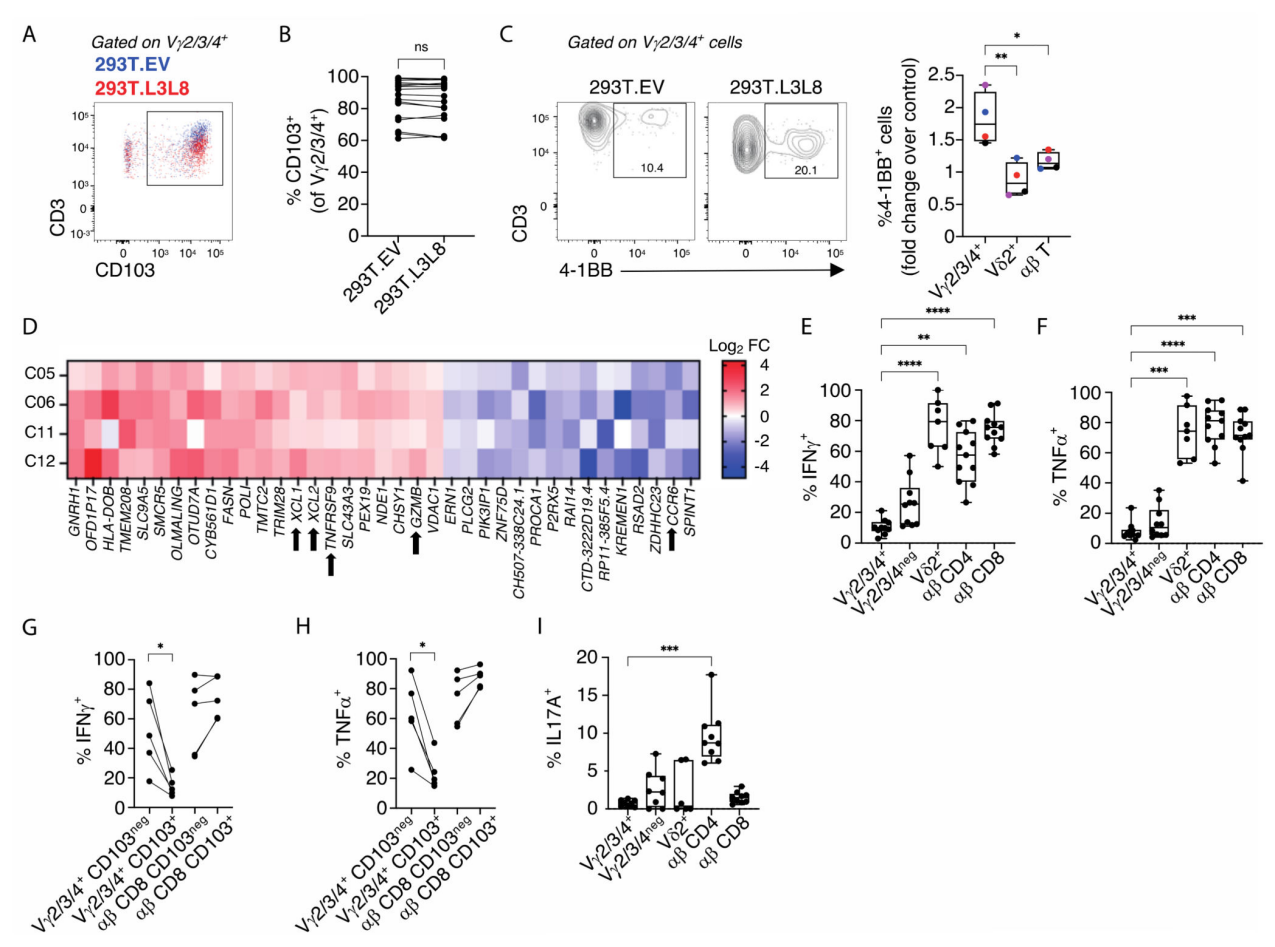
Vγ2/3/4^+^ cells make functional responses to BTNL3+BTNL8 stimulation but do not make classical effector cytokine responses. **(A).** Example flow plot of CD3 expression neg on CD103^neg^ and CD103^+^ Vγ2/3/4^+^ cells following overnight co-culture with 293T.EV (blue) or 293T.L3L8 cells (red). **(B).** Flow cytometric analysis of CD103 expression on intestinal Vγ2/3/4^+^ cells from control donors after co-culture as in A (n=18). Connected symbols represent individual participants. Paired *t*-test, ns – not significant **(C).** Flow cytometric analysis of 4-1BB expression on indicated colonic cell subsets (x axis) after co-culture as in A. Example flow plots (left) and summary graph (right) shown (n=4). Median, IQR and min-max range shown. Coloured symbols represent matched frequencies from individual participants. Repeated measures one-way ANOVA with Dunnett’s correction against Vγ2/3/4^+^ cells was used for analysis, *p<0.05; **p<0.01. **(D).** Significantly differentially expressed genes (x axis) in Vγ2/3/4^+^ cells co-cultured as in A. Data expressed as Log_2_ fold change (FC) for each donor (y axis). **(E,F).** Flow cytometric analysis of (E) IFNγ and (F) TNFα expression by the indicated colonic cell types (x axis) from control donors (n=7 -11) after 4h P+I stimulation. Median, IQR and min-max range shown. Symbols represent individual + participants. Kruskal-Wallis test with Dunn’s correction against Vγ2/3/4^+^ cells was used for analysis, **p<0.01; ***p<0.001; ****p<0.0001. **(G,H).** Flow cytometric analysis of (G) ++ TNFα and (H) IFNγ production by Vγ2/3/4^+^ γδ and CD8^+^ αβ T cells (x axis) (n=5) and split by their CD103 expression (legend) following 4h of P+I stimulation. Connected symbols represent individual participants. Multiple paired *t*-tests with Bonferroni-Dunn correction, *p<0.05. **(I).** Flow cytometric analysis of IL17A expression as in E and F (n=6-9). Median, IQR and min-max range shown. Symbols represent individual participants. Kruskal-Wallis + with Dunn’s correction against Vγ2/3/4^+^ cells was used for analysis, ***p<0.001.

We next asked how gut CD103^+^cells might respond to selecting and activating stimuli, respectively. As previously described (3), cells stained by an anti-Vγ2/3/4 antibody showed partial yet significant TCR downregulation (reduced gMFI of CD3) when co-cultured with HEK293T cells transduced to express BTNL3+BTNL8 (293T.L3L8) versus empty-vector transduced HEK293T cells (293T.EV) (fig S2A). There was considerable inter-individual variation (fig. S2A, B), but as expected, BTNL3+BTNL8 responsiveness (assessed by TCR downregulation) correlated with the representation of productive *TRGV4* rearrangements and correlated inversely with *TRGV2* rearrangements (fig. S2C). As evidenced by flow cytometry, TCR levels on CD103^+^ cells were higher relative to those on CD103^neg^ cells and were disproportionately downregulated by BTNL-exposure (Fig. 2A).

Of note, CD103 staining was unaffected by BTNL3+BTNL8 engagement (Fig. 2B), whereas many cells showing TCR downregulation upregulated the co-stimulatory receptor, TNFRSF9 (4-1BB) (Fig. 2C). Predictably, no response to BTNL3+BTNL8 was shown by V γ2^+^ or TCRαβ^+^ T cells in the same cultures (Fig. 2C). *TNFRSF9* transcript upregulation was also noted in an RNA sequencing study that was conducted to better understand the impact of BTNL-mediated selection on human Vγ2/3/4^+^Vγ2^neg^ T cells (Fig. 2D). Specifically, RNA was compared from 4 independent donors’ colonic T cells co-cultured with either 293T.L3L8 cells or 293T.EV cells (fig. S2D), revealing that in addition to *TNFRSF9*, there was upregulation of several genes including *XCL1, XCL2* and *GZMB* in contrast to which *CCR6* was downregulated (Fig. 2D). Collectively, these gene expression changes strongly phenocopy the response of murine intraepidermal Vγ5^+^ IEL responding to Skint1, their counterpart BTNL selecting element (36). Thus, CD103^+^Vγ4^+^ gut γδ T cells respond strongly and in an evolutionarily conserved fashion to BTNL3+BTNL8 engagement of their TCR, with conspicuous changes in expression of coregulatory receptors, chemokines and other effector potentials.

This notwithstanding, BTNL engagement of Vγ2^neg^ cells induced neither CD107a, a surrogate marker for induced cytolytic activity (fig. S2E), nor appreciable amounts of cytokines, e.g., TNFα, IFNγ, IL2 (fig. S2F, G). Furthermore, gut Vγ2^neg^ cells were strikingly hyporesponsive to PMA + ionomycin (P+I), a potent activator of signal transduction downstream of the TCR, by contrast to overt TNFα, IFNγ, and IL2 production induced in gutTCRαβ^+^ cells in the same cultures (fig. S2F, G). Indeed, Vγ2/3/4^+^ cells were the least responsive of gut T cells, and by paired analysis, CD103^+^Vγ2/3/4^+^ cells were less responsive than CD103^neg^Vγ2/3/4^+^ cells. By contrast, CD103 did not functionally segregate intestinal TCRαβ^+^ cells (Fig. 2G, H). Finally, whereas IL17A production was observed for ~10% of gut TCRαβ^+^CD4^+^ cells, it was only rarely displayed by Vγ2/3/4^+^ cells (Fig. 2I). In short, the yS compartment of healthy human gut includes CD103^+^ Vγ4Vγ1^+^ cells which respond strongly to TCR-mediated selection signals, provided by BTNL3+BTNL8, but which are recalcitrant to potent activating stimuli, specifically P+I.

### Gut Vγ4^+^ cells are unique

Focussing now on CD103^+^Vγ4^+^ T cells, we sought to elucidate the potential impact of BTNL3+BTNL8 on the signature phenotypes of human gut TCRyS^+^ T cells. To facilitate this, we used phage-display to obtain Vγ4-specific antibodies (see Methods), initially screening for reactivity to recombinant Vγ4, but not to recombinant Vγ2 (the V-region most similar to Vγ4). To identify Vγ4-specific antibodies unaffected by Vγ chain usage, and to epitope-map them, we screened for reactivity against human JRT3 cells transduced with various wild-type, mutated, and chimeric TCRs and, based on this, chose to use two antibodies (G4_18 and G4_9) (fig. S3A).

**Figure 3:**
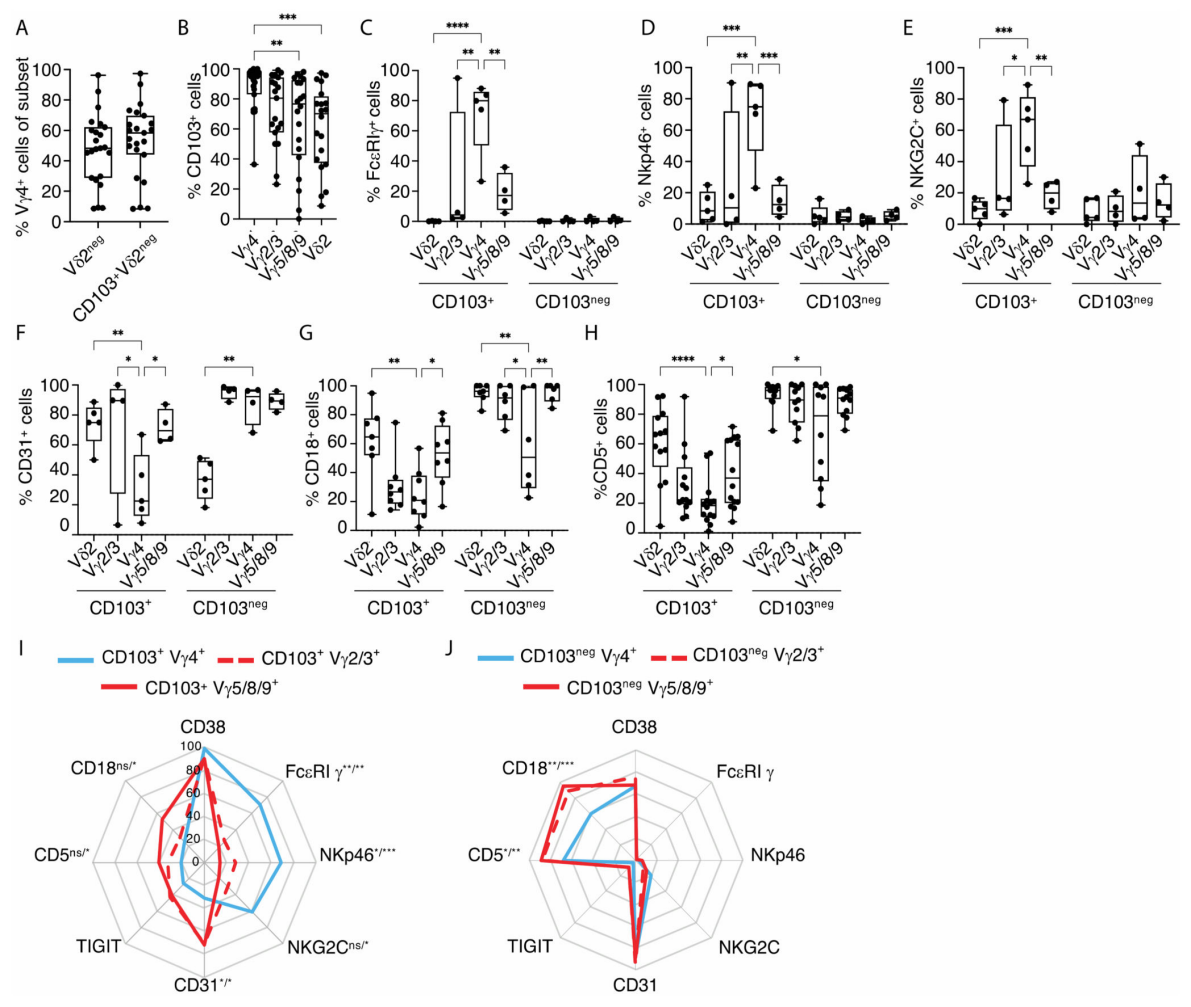
Vγ4 cells are phenotypically distinct from other γδ cells **(A).** Flow cytometric analysis of %Vγ4^+^ cells (y axis) in the indicated γδ subsets (x axis) from control biopsies (n=23). **(B).** Flow cytometric analysis of CD103 expression on the indicated γδ subsets (x axis) from control biopsies (n=19-23). Kruskal-Wallis test with Dunn’s correction against Vγ4^+^ cells was used for analysis, **p<0.01; ****p<0.0001. **(C-H)**. Flow cytometric analysis of (C) % FcεRIγ^+^ (n=4-5); (D) % Nkp46^+^ (n=4-5); (E) % NKG2C^+^ (n=4-5); (F) % CD31^+^ (n=4-5); (G) % CD18^+^ (n=6-8); (H) % CD5^+^ (n=10-14) cells on the indicated γδ subsets (x axis, CD103^+^ and CD103^neg^) from control gut tissue. Two-way ANOVA with Dunnett correction against Vγ4^+^ cells was used for analysis, *p<0.05; **p<0.01; ***p<0.001;****p<0.0001. (**A-H**) Median, IQR and min-max range shown. Symbols represent individual participants. **(I,J).** Radar plot of (I) the surface phenotype of CD103^+^ Vγ4^+^ versus CD103^+^ Vγ2/3^+^ versus CD103^+^ Vγ5/8/9^+^ γδ T cells; and (J) CD103^neg^ Vγ4^+^ versus CD103^neg^ Vγ2/3^+^ versus CD103^neg^ Vγ5/8/9^+^ γδ T cells from control donors (n=4-14). Plotted values represent mean of included participants. Two-way ANOVA + with Dunnett correction against Vγ4 cells was used for analysis, *p<0.05; **p<0.01; ***p<0.001; ns – not significant. Significance displayed as “versus Vγ2/3^+^ / versus + Vγ5/8/9^+^”.

In control samples of colonic Vγ2^neg^ T cells, the mean frequency staining for Vγ4^+^ cells was 47.7% (Fig. 3A), although as was observed for *TRGV4* transcript reads (see Fig. 1, above), there was high inter-individual variation, with Vγ4^+^ lymphocytes often failing to compose the majority of yS cells. The fact that the median value for Vγ4^+^ cells of 48.3% [95% CI 29.8-60.1; n=23] exceeds the median *TRGV4* reads (24.26% [95% CI 18.8-30; n=13]) most likely reflects the respective denominators, i.e., Vγ2^neg^ T cells versus whole gut biopsies used for the transcript reads, and greater purity of the flow cytometry samples. Investigating further, it was the CD103^+^Vγ2^neg^ cell subset that was relatively enriched in Vγ4^+^ cells (Fig. 3A), consistent with which, a greater majority of Vγ4^+^ cells were CD103^+^, by comparison with Vγ2/3^+^, Vγ5/8/9^+^ and Vγ2^+^ cells (Fig. 3B), strongly suggesting a greater propensity of Vγ4^+^ cells to occupy intraepithelial niches.

Next, to assess whether intestinal Vγ4^+^ T cells displayed unique traits that might be attributable to BTNL-mediated interactions, we used an anti-Vγ4 antibody (G4_18; above) together with existing antibodies to purify cells for RNASeq from four donors. To maximise potential differences, we first compared Vγ4^+^ with Vγ5/8/9^+^ cells, and then examined differentially expressed genes across the broader range of γδ T cells. In fact, there were very few significant differences, but attesting to the dataset’s validity, *TRGV4* was the most prominently enriched RNA in Vγ4^+^ cells, while *TRGV5* was significantly under-represented (table S3). Beyond *TRGV4*, the most differentially expressed RNA was *FCER1G*, encoding FcεR1γ, a protein that can pair with and/or substitute for CD3ζ in TCR-induced signal transduction (37, 38), particularly in mouse intestinal IEL (39), and that can transduce signals from NK receptors (see below). We noted that RNA encoding *API5*, recently implicated in protecting Paneth cells (11), was not significantly overexpressed in Vγ4^+^ cells relative to other γδ T cells.

Whereas the transcriptomic analysis was undertaken on four donors, validation was sought using flow cytometry on many more independent donors. Illustrative plots presented with graphs of multiple donor data validated that FcεR1γy was highly enriched in gut CD103^+^Vγ4^+^cells versus cells expressing any other Vγ regions, except for a single donor in which it was highly expressed by CD103^+^Vγ2/3^+^ Vγ4^neg^ cells (Fig. 3C; fig S3B). Strikingly, there was no expression of FcεR1γy in any subset of CD103^neg^γδ T cells (Fig. 3C), nor in any TCRVγ-subtype in PBMC, including Vγ4^+^ cells (fig. S3C). Hence, FcεR1γy is in essence a signature phenotype of cells defined by CD103 expression, implying epithelial association; by intestinal or more general extra-lymphoid localisation; and by specific usage of TCRVγ4 that uniquely facilitates BTNL3+BTNL8-responsiveness. Interestingly, this same pattern proved applicable to some NK receptors, e.g., NKp46 and NKG2C (Fig. 3D, E; fig. S3D, E). Conversely, intestinal CD103^+^Vγ4^+^ cells showed significantly lower expression of an adhesion molecule, CD31 *(PCAM1)* (Fig. 3F; fig. S3F), by contrast to significantly higher expression of its ligand, CD38 (fig. S3G).

Likewise, whereas *CD18* and *CD5* were less frequently expressed by CD103^+^ versus CD103^neg^ yS cells (see Fig 1I, above), they were expressed at disproportionately low levels by CD103^+^Vγ4^+^ cells (Fig. 3G, H). Thus, by several criteria, Vγ4^+^CD103^+^ gut T cells displayed a unique phenotype by comparison to CD103^+^ gut T cells expressing other Vγ chains (Fig. 3I), thereby attesting to a profound impact of interactions with BTNL3+BTNL8. At the same time, gut CD103^neg^Vγ4^+^cells were more similar to CD103^neg^ cells expressing other Vγ chains (Fig. 3J), which suggests that either there are modifiers of the impact of BTNL-mediated selection on Vγ4^+^cells, or that selection of CD103^neg^Vγ4^+^cells was incomplete, possibly because the cells were in transition to the epithelium.

### Disproportionate CD103^+^Vγ4 cell loss in inflammatory bowel disease

**Figure 4:**
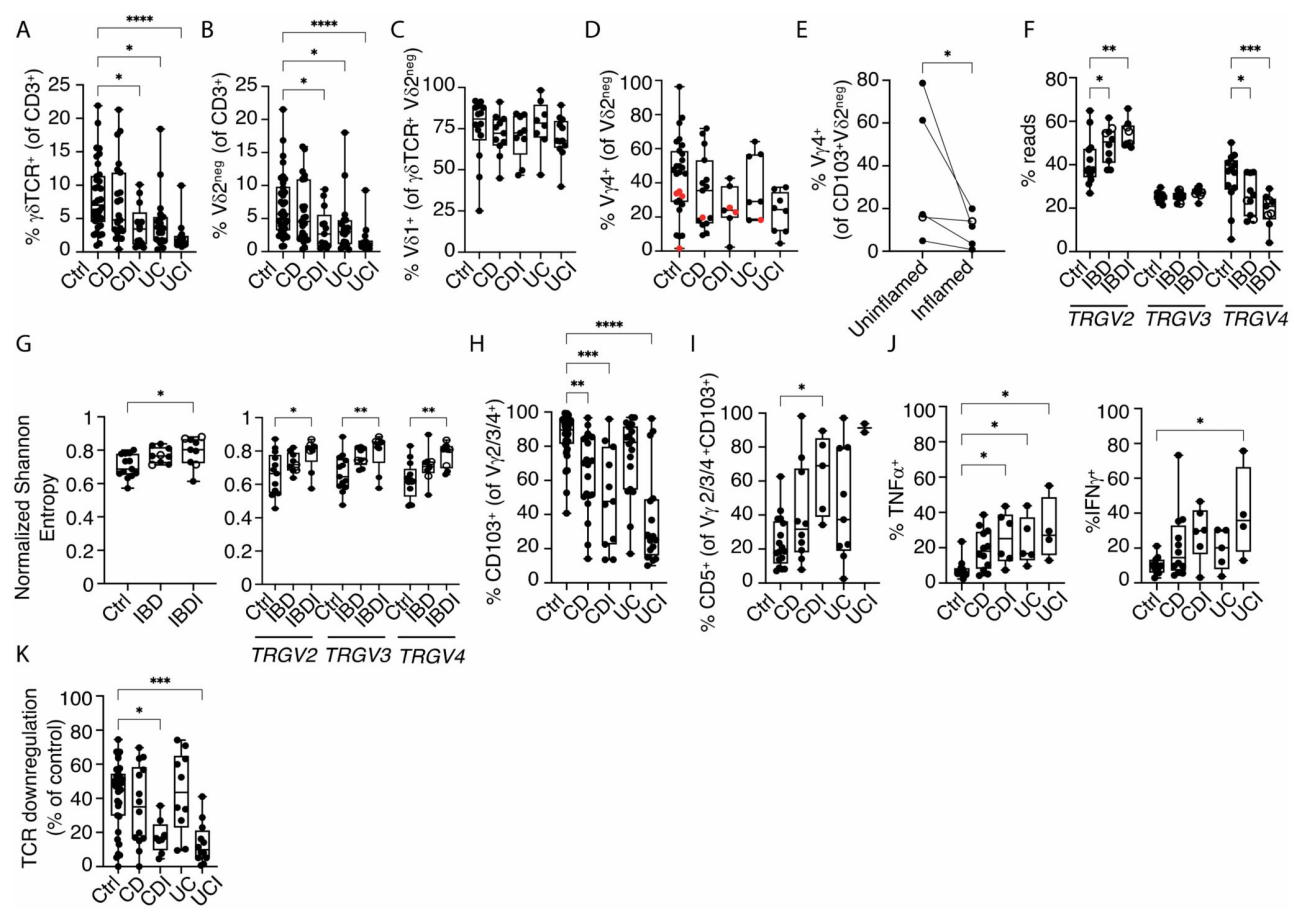
Phenotypic and clonotypic changes of the intestinal γδ T cells compartment in IBD. **(A-C).** Flow cytometric analysis of (A) % γδTCR^+^, and (B) % Vδ2^neg^ cells of total T lymphocytes isolated from control (n=34), CD (n=23), CDI (n=13), UC (n=19) and UCI (n=14) donors; and (C) % Vδ1^+^ of Vδ2^neg^ γδ T cells isolated from control (n=14), CD (n=11), CDI (n=9), UC (n=8) and UCI (n=12) donors. Kruskal-Wallis test with Dunn’s correction against control donors was used for analysis, *p<0.05; ****p<0.0001. **(D).** Flow cytometric analysis of % Vγ4^+^ cells of Vδ2^neg^ colonic γδ T cells from control (n=27), CD (n=15), CDI (n=7), UC (n=7), UCI (n=8) biopsies. Red dots – BTNL genotype discussed subsequently. **(E).** Flow cytometric analysis of % Vγ4 cells of CD103^+^ Vδ2^neg^ colonic γδ T cells from paired uninflamed and inflamed tissue from IBD donors sampled at the same endoscopy (n=5). Connected symbols represent individual participants. Open circles – CD donor; Filled circles – UC donors. Ratio paired *t*-test, *p<0.05. **(F).** *TRGV2, TRGV3, TRGV4* gene usage as proportion of total *TRGV2/3/4* reads from TCR deep sequencing of whole biopsies mRNA (Ctrl n=13; IBD n=9, IBDI n=9). Open circles - CD donors; filled circles - UC donors. Twoway ANOVA with Dunnett correction against control donors was used for analysis, *p<0.05; **p<0.01. **(G).** Total γδTCR (left) and *TRGV2/3/4* (right) diversity measured by normalized Shannon entropy derived from TCR deep sequencing of whole biopsies mRNA from control (n=13), IBD (n=9) and IBDI (n=9) donors. Open circles – CD donors; filled circles – UC donors. Two-way ANOVA with Dunnett correction against control donors was used for analysis, *p<0.05; **p<0.01; ***p<0.001. **(H).** Flow cytometric analysis of CD103 expression on Vγ2/3/4^+^ colonic γδ T cells derived from control (n=43), CD (n=22), CDI (n=11), UC (n=18), UCI (n=17) donors. Kruskal-Wallis test with Dunn’s correction against control donors was used for analysis, **p<0.01; ***p<0.001; ****p<0.0001. **(I).** Flow cytometric analysis of CD5 expression on CD103^+^ Vγ4^+^ colonic γδ T cells derived from control (n=17), CD (n=10), CDI (n=5), UC (n=9), UCI (n=2) donors. Kruskal-Wallis test with Dunn’s correction against control donors was used for analysis, *p<0.05. **(J).** Flow cytometric analysis of TNFα (left) and IFNγ (right) expression by Vγ2/3/4^+^ colonic lymphocytes obtained from control (n=11), CD (n=12), CDI (n=6), UC (n=5) and UCI (n=4) donors following 4h of P+I stimulation. Kruskal-Wallis test with Dunn’s correction against control donors was used for analysis, *p<0.05. **(K).** Flow-cytometric analysis of %TCR downregulation by human colonic Vγ2/3/4^+^ lymphocytes derived from control (n=34), CD (n=14), CDI (n=8), UC (n=10) and UCI (n=12) donors, after overnight co-culture with 293T.EV or 293T.L3L8 cells. Kruskal-Wallis test with Dunn’s correction against control donors was used for analysis, *p<0.05; ***p<0.001. All box and whisker plots: median, IQR and min-max range shown. Symbols represent individual participants.**+**

We next investigated how the gut γδ T cell compartment, and within it the unique CD103^+^ Vγ4^+^ subset, might be affected in patients with IBD, by sampling inflamed (IBDI) and macroscopically uninflamed regions (IBD). Whether sampling inflamed Crohn’s Disease (CDI) or inflamed ulcerative colitis (UCI), yS cell frequencies in IBDI were significantly less than in outwardly healthy gut (Fig. 4A), and this was also observed for uninflamed regions of UC (Fig. 4A). While the representation of Vγ2^+^ cells among colonic T cells (ordinarily the minor subset [see fig S1A, above]) was also reduced in CDI, UC and UCI (fig. S4A), there were more overt reductions of Vγ2^neg^ cell frequencies, again being significant in each of UC, UCI, and CDI (Fig. 4B). Furthermore, whereas Vγ2^neg^ cells remained mostly Vγ1^+^ (Fig. 4C), the frequencies of Vγ4^+^ cells in IBDI were reduced, in both CDI and UCI (Fig. 4D; note that red circles denote 8 donors with distinct BTNL genotypes, to be considered below). Moreover, significant disease-associated reductions in the frequency of Vγ4^+^ cells in the CD103^+^Vγ2^neg^ compartment were evident when paired inflamed sites were compared to uninflamed sites from the same donors (Fig. 4E; CD sample, open circle; and UC samples, filled circles). This was further corroborated by deep sequencing which showed significant, selective reductions in *TRGV4* relative to increased *TRGV2* within the Vγ2/3/4^+^ compartment (Fig. 4F; CD samples, open circles; UC samples, filled circles).

In going from controls through IBD to IBDI, the overall Vγ repertoire and the Vγ2, Vγ3, and Vγ4 sub-repertoires all showed increasing TCR sequence diversity as measured by Shannon entropy, reaching significance in the control versus IBDI groups, even when there was no aggregate change in representation, i.e., *TRGV3* (above) (Fig. 4G). This lack of TCR focussing strongly implied that the Vγ repertoire shifts that included the relative loss of Vγ4^+^ cells, were not driven by clonotypic antigens, unlike the clonal dynamics reported for Vγ1^+^ cells in other settings, e.g., response to cytomegalovirus (40, 41). This nonclonal, innate-like response of total δγ and the Vγ2, Vγ3, and Vγ4 sub-repertoires was validated for IBDI by D50, an independent measure of diversity (fig. S4B).

We therefore considered that the repertoire changes might reflect cells being recruited to the gut de novo, consistent with which there was an increase in proportion of Vγ2^neg^ cells that were CD103^neg^ and that co-expressed CD45RA and CD27 which jointly demarcate poorly differentiated cells. (fig. S4C, D) (42). Indeed, there was more generally a significant reduction from ~90% to ~55% of Vγ2/3/4^+^Vγ2^neg^ cells expressing CD103 in CDI, and from ~90% to ~30% in UCI (Fig. 4H), albeit with inter-individual variation, and this was well illustrated for Vγ4^+^Vγ2^neg^ cells by comparing paired inflamed and uninflamed regions from the same individuals sampled at the same endoscopy (fig. S4E, F). Additionally, in a subset of donors where the data were available, the residual CD103^+^ Vγ2/3/4^+^ T cells in UCI and CDI showed atypically high frequencies of CD5^+^ cells (Fig. 4I) which in the healthy colon is a phenotype more often associated with CD103^neg^Vγ2/3/4^+^ cells as considered above (see Fig. 1F). Hence, the main dysregulation in IBD was a shift away from the signature frequency and phenotype of CD103^+^Vγ4^+^ *cells*.

Because we had observed that among T cells in the healthy colon the CD103^+^Vγ2/3/4^+^Vγ2^neg^ subset was the most refractory to strong activation, we considered that the relative increases in CD103^neg^ cells in IBDI donors as presented above (see Fig. 2E, F) might translate into precocious cytokine production. Indeed, donors with CDI, UC and UCI showed significantly higher frequencies of Vγ2/3/4^+^ cells producing TNFα and, for UCI, IFNγ in response to P+I (Fig. 4J). There were no significant increases in IL17A-producing Vγ2/3/4^+^ cells (fig. S4G). Moreover, of several gut T cell subsets sampled, only Vγ2/3/4^+^ cells showed significantly increased frequencies of cytokine producing cells in IBDI, although it is important to note that in each case, those frequencies were substantially less than the baseline frequencies of cytokine-producing Vγ2^+^ and αβ T cells in both the healthy and diseased colon (Fig. 4J; fig. S4G, H, I). Hence, increased inflammatory cytokine production by Vγ2/3/4^+^ cells may be more profound in its reflection of the cells’ altered phenotype rather than any substantial contribution to local cytokine levels. Indeed, paralleling their increased effector responses, Vγ2/3/4^+^ cells showed significantly decreased responses to 293T.L3L8 (Fig. 4K), consistent with the reductions in Vγ4^+^ cells. We concluded that in many individuals with IBD, particularly in inflamed lesions, there was a disproportionate diminution and alteration of the unique colonic CD103^+^Vγ4^+^ cell compartment, which is in turn associated with increased responses of the residual cell pool to an activating stimulus and reduced responses to a selecting stimulus. Therefore, we focussed next on the prospect that a major deleterious factor in IBD was the reduction in signature CD103^+^Vγ4^+^ cells. This would echo the severe exacerbation of murine colitis in the absence of γδ T cells that ordinarily promote tissue repair and homeostasis (8).

### Inflammatory bowel disease-associated cytokines dysregulate CD103^+^Vγ4^+^ T cells

Initially we asked what factors might induce the depletion and/or altered phenotypes of intestinal Vγ4^+^CD103^+^ cells observed in IBDI. Whilst those altered phenotypes were suggestive of newly infiltrating CD103^neg^CD45RA^+^CD27^+^ γδ cells (see fig. S4C, D), we also hypothesised that the IBD milieu might contribute. It is challenging to quantitate gene expression in diseased versus control whole gut preparations because cell compositions and phenotypes can change dramatically. Nonetheless, the data revealed significantly increased transcripts encoding the cytokines IL12, IL18, IL23, and TNFα in IBDI (fig. S5A; note that although CD samples (open circles) and UC samples (closed circles) were pooled as IBD or IBDI, their data points completely overlapped). These cytokines are germane to IBD because of their proinflammatory nature and because antibodies neutralising TNFα, IL12/IL23, and IL23 are all efficacious in IBD (43–45). Additionally, Vγ4 cells express RNAs encoding receptors for IL12, IL18, and TNFα (fig. S5B). Thus, we cultured primary gut T cells in IL2+IL15 for 7 days, additionally supplementing with IL12+IL18, or TNFα, or IL1β+IL23 (which might induce changes in Vγ4 cells indirectly *via* cells expressing the cognate receptors).

**Figure 5:**
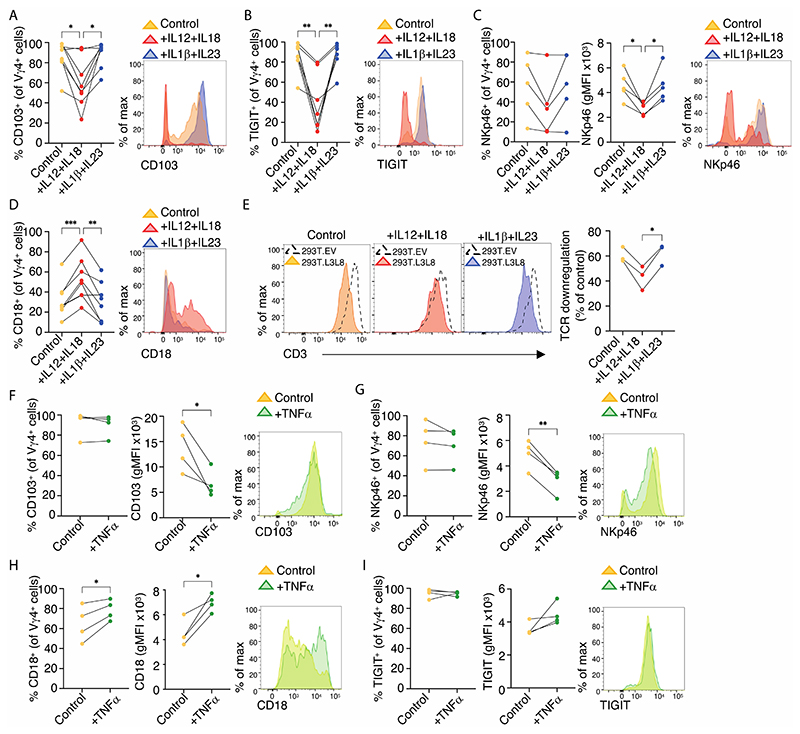
The cytokine milieu can affect the phenotype of intestinal Vγ4^+^ cells. **(A-D).** Flow cytometric analysis of (A) CD103 (n=8), (B) TIGIT (n=8), (C) NKp46 (n=5), and (D) CD18 (n=8) expression on Vγ4^+^ colonic lymphocytes cultured for 7 days in IL2+IL15 (control); IL2+IL15+IL12+IL18 and IL2+IL15+IL1β+IL23. Summary data (left) and representative histograms (right) shown. Connected symbols represent individual participants. Repeated measures one-way ANOVA with Dunnett’s correction against IL12+IL18 treatment was used for analysis, *p<0.05; **p<0.01; ***p<0.001. **(E).** Flow cytometric analysis of Vγ2/3/4^+^ colonic lymphocytes cultured for 7 days as in A-D and subsequently co-cultured overnight with 293T.EV and 293T.L3L8 cells (n=3). Representative flow plots of CD3 expression and summary graph (far right) of percent TCR downregulation relative to 293T.EV control are shown. Repeated measures one-way ANOVA with Bonferroni correction, *p<0.05. **(F-I).** Flow cytometric analysis of (F) CD103, (G) NKp46, (H) CD18 + and (I) TIGIT expression on Vγ4 colonic lymphocytes cultured for 7 days in IL2+IL15 (control) and IL2+IL15+TNFα (n=4). Summary data (left, centre) and representative histograms (right) shown. gMFI - geometric mean fluorescent intensity. Connected symbols represent individual participants. Paired *t*-test, *p<0.05; **p<0.01.

Strikingly, IL12+IL18 selectively shifted Vγ4^+^ cells away from the signature healthy gut phenotype, showing instead significant reductions in the frequencies of cells expressing CD103 and TIGIT (Fig. 5A, B) and significantly reduced expression levels of NKp46 (Fig. 5C). Complementing these changes, there was an increased frequency of cells expressing CD18 (Fig. 5D) that is ordinarily associated with CD103^neg^ cells (see Fig. 1F). Consistent with this overall phenotypic change, Vγ2/3/4^+^ cells showed less TCR downregulation in response to BTNL3+BTNL8, although it is also the case that their TCR expression was lower at the outset (Fig. 5E). Conversely, no changes were observed in response to IL1β+IL23 (Fig. 5A-E). Interestingly, TNFα exposure did not reduce the frequencies of cells expressing CD103 or NKp46 but it significantly reduced CD103 and NKp46 cell surface expression levels, and it also increased both the frequencies of cells expressing CD18 and its surface expression levels (Fig. 5F, G, H), while having no clear impact on TIGIT (Fig. 5I). Hence, one can conclude that the signature CD103^+^Vγ4^+^ gut T cell phenotype might not only be displaced by infiltrating cells (see fig. S4C, D), but that it is vulnerable to the selective actions of cytokines characteristic of the IBD milieu. Therefore, the prospect exists that the signature CD103^+^Vγ4^+^ gut T cell phenotype might also be disrupted by other factors of the IBD milieu that we have not tested, including reported reductions in BTNL3+BTNL8 expression (15, 46).

### BTNL3+BTNL8 are selection/maintenance factors for intestinal Vγ4^+^ T cells

**Figure 6:**
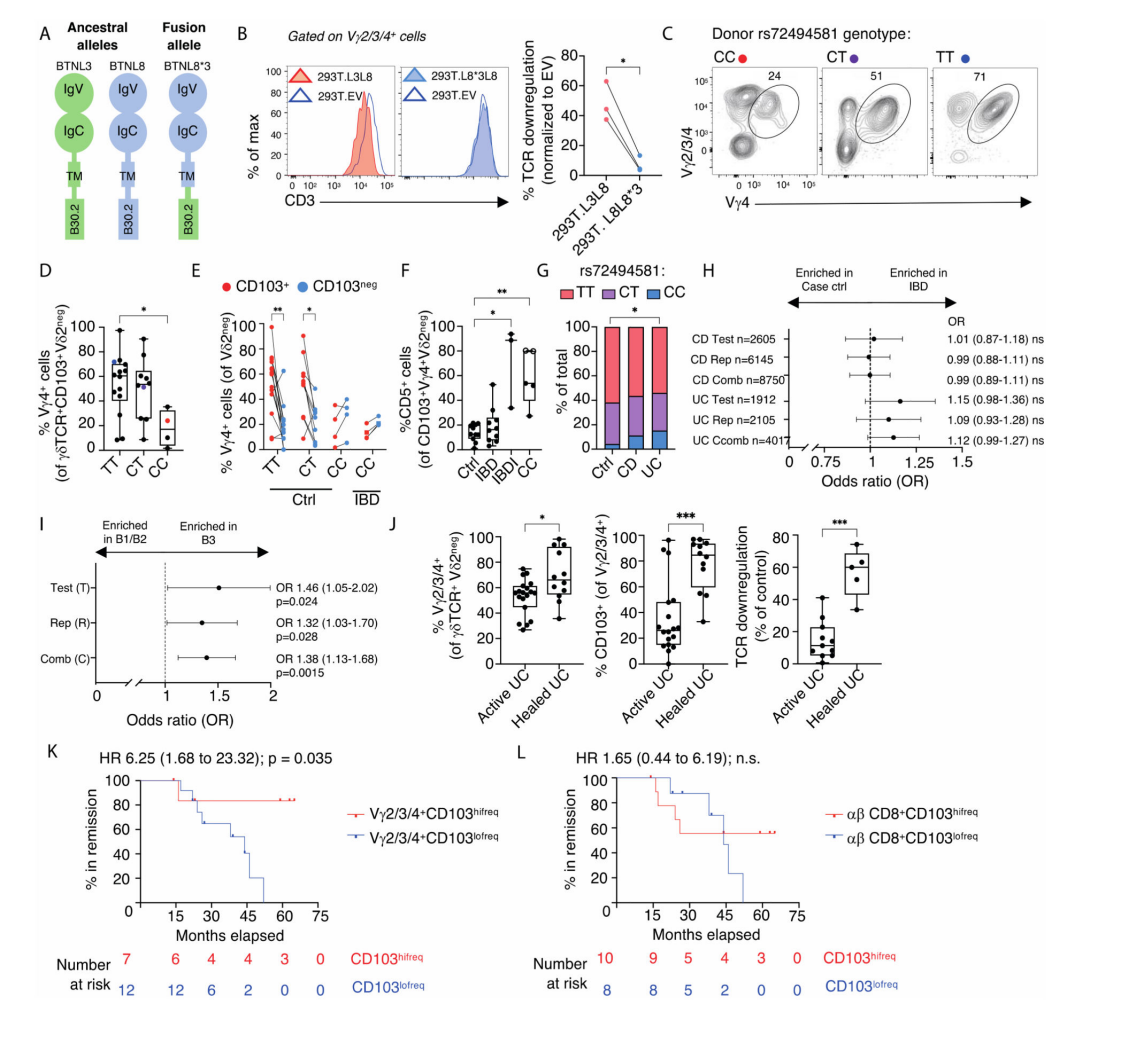
A genetic influence on the Vγ4-BTNL axis. **(A).** Graphical representation of full length BTNL3, BTNL8 and BTNL8*3 fusion protein structure. TM – transmembrane. **(B).** Flow cytometric analysis of human colonic Vγ2/3/4^+^ cells co-cultured overnight with 293T.EV, 293T.L3L8 and 293T. L8L8*3 (n=3). Example flow plots of CD3 expression and summary graph (far right) of %TCR downregulation relative to 293T.EV control are shown. Connected symbols represent individual participants. Paired *t*-test, *p<0.05. **(C).** Example neg flow cytometry plots of % Vγ4^+^ cells of CD103^+^ Vγ2^neg^ γδ T cells obtained from control biopsies. rs72494581 status indicated above plots. Coloured dots correspond to data points neg displayed in panel D. **(D).** Flow cytometric analysis of % Vγ4^+^ cells of CD103^+^ Vγ2^neg^ γδ T cells according to their rs72494581 SNP genotype in control donors. CC (n=4), CT (n=10), TT (n=14). Median, IQR and min-max range shown. Symbols represent individual participants. Kruskal-Wallis test with Dunn’s correction against control donors was used for neg analysis, *p<0.05. **(E).** Flow cytometric analysis of % Vγ4^+^ cells (y axis) of CD103^+^ Vγ2^neg^ (red) or CD103^neg^ Vγ2^neg^ (blue) populations in control donors with different rs72494581 genotype (x axis) and IBD donors with the CC genotype. Square – UC donor. CC Ctrl (n=4),CC IBD (n=4), CT Ctrl (n=10), TT Ctrl (n=14). Connected symbols represent individual participants. Wilcoxon matched-pair test, *p<0.05; **p<0.01. **(F).** Flow cytometric analysis of % CD5^+^ cells of Vγ4^+^ CD103^+^ Vδ2^neg^ lymphocytes from control (n=11), IBD (n=10) and IBDI (n=3) TT and CT donors, and control (n=3) and uninflamed IBD (n=2, open circles) CC donors. Median, IQR and min-max range shown. Symbols represent individual participants. Kruskal-Wallis test with Dunn’s correction against control donors was used for analysis, *p<0.05; **p<0.01. **(G).** Frequency of the rs72494581 genotype within the local cohort (ctrl n=115 ; CD n=64 ; UC n=52). Fisher’s exact test, *p<0.05. **(H).** Odds ratios (OR) for development of CD or UC compared to 1950s birth cohort data. OR (95% confidence intervals) presented according to the minor allele (CC) and risk of developing disease. **(I).** OR for development of B3 versus B1/B2 CD. OR (95% confidence intervals) presented according to the minor allele (CC) and risk of developing disease complications (T n=1817; R n=4491; C n=6308). H,I. Test - test set; Rep – replication set; Comb – combined set. **(J).** Flow cytometric analysis of: percent Vγ2/3/4^+^ cells (left); % CD103^+^ Vγ2/3/4^+^ cells (middle); and % TCR downregulation by γδ lymphocytes after overnight co-culture with 293T.EV or 293T.L3L8 cells (right) in the same donors. Colonic biopsies were obtained from inflamed (n=11 -19) and previously inflamed (healed) areas (n=5-12). Median and inter-quartile range shown (box) with minimum and maximum values (whiskers). Symbols represent individual participants. Mann-Whitney U test, *p<0.05; ***p<0.001. **(K,L).** Hazard ratio (HR) analysis of IBD patients in remission with intestinal (K) CD103^hifreq^ Vγ2/3/4^+^ cells (CD n=3; UC n=4) and CD103^lofreq^ Vγ2/3/4^+^ cells (CD n=8; UC n=4); and (L) CD103^hifreq^ CD8^+^ αβ T cells (CD n=4; UC n=6) and CD103^lofreq^ CD8^+^ αβ T cells (CD n=6; UC n=2) in colonic biopsies at time of sampling. Subjects followed until the end of the study or relapse. Log rank test, Hazard ratio log rank, ns – not significant.

There are several challenges in investigating the potential impacts on CD103^+^Vγ4^+^ gut T cells of reported reductions in BTNL3+BTNL8 expression in whole IBD gut. Specifically, as was considered above in the context of cytokine expression, there are changes in cell composition and activation states, added to which there has been no measure of whether surface BTNL3+BTNL8 expression by colonocytes adjacent to Vγ4^+^ T cells is de facto reduced. Therefore, to unequivocally assess such a situation, we turned to a well-established copy number variation (CNV) polymorphism in which deletion causes an in-frame fusion of the BTNL8 N-terminus to the BTNL3 C-terminus (Fig. 6A) (47). Homozygosity for this allele occurs at ~9% in Europeans. The allele could be identified by a standard PCR assay (fig. S6A) but given that CNVγ can be challenging to detect by routine genetic analyses, we also identified a surrogate SNP rs72494581 to facilitate reliable identification of the CNV in DNA microarrays (fig. S6B, C; Methods). From this analysis, CNV homozygotes derived their annotation, “CC”, compared to “TT”, denoting homozygosity for the high frequency allele (full length *BTNL8* and *BTNL3)* and “CT”, denoting heterozygotes (fig. S6C).

When the “C” allele was cloned, FLAG-tagged, and expressed in HEK293T cells, its protein product (BTNL8*3) showed poor cell surface expression (fig. S6D, bottom left). We previously reported BTNL8 co-expression can rescue poor surface BTNL3 expression (3, 48), but by contrast, BTNL8*3 was ineffective at this (fig. S6D, 2^nd^ row). Moreover, whereas BTNL3 can enhance surface BTNL8 expression (fig. S6D, 3^rd^ row, left and centre), it was less effective at rescuing BTNL8*3 expression (fig. S6D, 4^th^ row, left and centre). Added to this inefficient cell surface expression, BTNL8*3 lacks the BTNL3 N-terminal V-region that directly engages human Vγ4 (13), and reflecting this, BTNL8*3+BTNL8 could not drive surface Vγ4Vγ1 TCR downregulation (Fig. 6B). Thus, in relation to γδ T cell regulation, BTNL8*3 is encoded by a severely hypomorphic allele.

Because BTNL1+BTNL6 drive selection of the mouse intestinal Vγ7^+^ γδ cell compartment (3), BTNL1-deficient mice display severe, albeit incomplete losses of Vγ7^+^ IEL, while in BTNL6-deficient mice, Vγ7^+^ IEL are reduced by ~50% (4). Moreover, once the compartment forms, neither BTNL1 nor BTNL6 is necessary in the short-term to maintain Vγ7^+^ IEL, but they do maintain several parameters of the cells’ phenotype (4). In this regard, persons homozygous for BTNL8*3 offered an opportunity to ask whether analogous mechanisms regulated the human gut γδ T cell compartment. Indeed, frequencies of Vγ4^+^ gut T cells were significantly, albeit incompletely, reduced in healthy individuals with a CC genotype (Fig. 6C, D), that were also those denoted by red circles among the control cohort depicted in Fig 4D (above: Note, however, that even when removing *a posteriori* those individuals homozygous for CC from the overall comparison, there was still a significant reduction in Vγ4^+^ cell frequencies in IBDI (fig S6E)). As well as constituting a smaller fraction of the Vγ2^neg^ compartment in persons homozygous for the CC genotype, the residual Vγ4^+^ T cells were also phenotypically altered. Thus, whereas for donors with CT and TT genotypes, most Vγ4^+^ T cells were CD103^+^, this association of Vγ4 and CD103 was lost in homozygotes for BTNL8*3, who displayed comparable frequencies of CD103^+^ and CD103^neg^ Vγ4 cells (Fig. 6E).

The severed association of Vγ4 and CD103 phenocopied the shift from CD103^+^ to CD103^neg^ cells among Vγ4^+^Vγ2^neg^ cells in IBDI (shown above in fig. S4E). Indeed, we identified four IBD patients with the CC genotype who also showed reduced frequencies of Vγ4^+^ T cells (also denoted in red in Fig 4D, above) and the shift from CD103^+^ to CD103^neg^ Vγ4 cells (Fig. 6E). Moreover, CD103^+^Vγ4^+^ lymphocytes from CC donors further phenocopied IBDI in that they showed significantly higher CD5 expression (Fig. 6F). However, because most IBD patients we studied carried either the TT or CT phenotype, we could conclude that there were two distinct settings showing comparable diminution and phenotypic alteration of colonic CD103^+^Vγ4^+^ T cells: IBDI, irrespective of BTNL genotype, and BTNL loss-of-function, irrespective of disease status. Importantly, the reduction in frequency and altered phenotypes of CD103^+^Vγ4^+^ cells in persons homozygous for BTNL8*3 jointly establish that humans, like mice, deploy BTNL proteins to select and/or maintain cognate gut γδ T cells with a unique phenotype. That neither BTNL-deficient mice nor humans homozygous for BTNL8*3 showed complete loss of Vγ7^+^ and Vγ4^+^ gut T cells, respectively, is discussed below.

### BTNL3+BTNL8 hypomorphism is a disease modifier

A genetic basis for Vγ4 cell dysregulation in outwardly healthy individuals offered the opportunity to investigate whether this might predispose to disease. In a pilot snapshot assessment in our clinical practise, we noted that among 64 patients with CD, the frequency of CC exceeded that among 115 control donors, and that the CC frequency was significantly greater among 52 patients with UC (Fig. 6G). We therefore investigated the frequency of CC homozygotes in 12,767 individuals with IBD comprising a test cohort (n=4517) and a replication cohort (n=8250) provided by the UK Bio-resource, against population-based controls from the 1958 British Birth Cohort (n=6767) (49). Notwithstanding a trend in UC, the CC genotype showed no significant association with disease incidence (Fig. 6H). By contrast, when disease phenotype was assessed in 6308 patients with CD (Test 1817; Replication 4491), there was a significant association of CC frequency with severe “B3” disease which penetrates through the bowel wall forming intra-abdominal abscesses or fistulae (11.9%, 146/1225), compared with inflammatory (B1) or stricturing (B2) disease which remains confined within the intestinal lumen (8.7%, 455/5083) (Fig. 6I) (50). We conclude that for those with CD, BTNL3 loss-of-function and/or consequent dysregulation of gut Vγ4^+^CD103^+^ cells predispose toward penetrating disease and is therefore a disease modifying factor. The lack of a similar severity classification based on objective disease behavioural parameters precluded a similar analysis of UC.

In identifying the association with penetrating CD, we considered several potentially confounding issues. First, the test-set was an older cohort from tertiary referral centres, London and Newcastle, collected between 1999 and 2017 wherein B3 disease is represented at 24%, whilst the replication-set is the NIHR UK IBD bioresource cohort started in 2016, wherein only 16% are classified as B3, possibly reflecting improving therapeutic approaches, including anti-cytokine antibodies, more aggressive top-down strategies, early surgery, and standardisation of care through guideline development. Moreover, to mitigate the potential effect of disease duration on findings, we increased the stringency of our study by only including patients with 5-year follow up in the B1 cohort, although clearly this could exclude some patients who might later have transitioned to B3 disease (51). These considerations notwithstanding, our genetic analysis was still able to identify a significant association of BTNL3+BTNL8 / Vγ4^+^ T cell deficiency with penetrating CD.

### Inflammatory bowel disease treatment response associates with γδ T cell reversion

For the concluding part of our study, we investigated potential associations of gut γδ cells with responses to treatment. Unlike the characteristic skip lesions of CD, UC is characterised by confluent inflammation: thus, in persons with extensive colitis, one can be confident that samples of uninflamed colonic mucosa that lie toward the rectal side of the extent of disease will have previously been inflamed. Thus, we examined such areas of macroscopically healed mucosa from regions known to be previously affected and found that some such regions displayed recovery of the healthy gut phenotype by the criteria of the frequency of Vγ2/3/4^+^ cells among Vγ2^neg^ cells; the proportion of those Vγ2/3/4^+^ cells expressing CD103; and the TCR downregulation response to BTNL3+BTNL8 (Fig. 6J). Thus, in seeming contrast to reports of gut γδ T cell dysregulation in celiac disease (14), the nonclonal switch in the signature γδ T cell compartment observed in inflamed IBD lesions seemed to be reversible, evoking the wound healing process to which γδ T cells can contribute as reported in murine colitis (8-10, 52).

The capacity of histological assessments of healing to define IBD remission, particularly in Crohn’s disease, has remained unclear (53). We therefore examined whether the phenotypic renormalisation of the colonic γδ T cell compartment might be associated with sustained remission. To this end, we assessed the status of 19 patients who at time of sampling were in complete mucosal and clinical remission on treatment (table S4). The patients were classified as “Vγ2/3/4^+^CD103^hifreq^” if the percentage of their Vγ2/3/4^+^ cells expressing CD103 scored within the 95% confidence intervals of the median value of control CD103 expression by Vγ2/3/4^+^ cells (i.e., >83.5% [see Fig 1B, above]), and contrasted them with Vγ2/3/4^+^CD103^lofreq^ individuals who fell below that range. Of 19 patients, 7 (3 CD; 4 UC) scored as Vγ2/3/4^+^CD103^hifreq^ whereas 12 patients (8 CD; 4 UC) were Vγ2/3/4^+^CD103^lofreq^. When their disease progression was compared over the next five years, either until relapse or the end of the study, only one relapse was observed in the Vγ2/3/4^+^CD103^hifreq^ group,whereas 9/12 Vγ2/3/4^+^CD103^lofreq^ patients relapsed (hazard ratio = 6.25 (1.68-23.32 p=0.035)) (Fig. 6K). Conversely, 18 of the 19 patients collectively showed no reliable association of remission / relapse with the phenotype of colonic CD103^+^CD8^+^ αβ T cells (for one of the 19 patients, CD8 ^+^ T cell data could not be recovered for technical reasons) (Fig. 6L).

## Discussion

By investigating the human colonic T cell compartment, we have combined human genetics with tissue immunological studies to establish that the BTNL-dependent selection and phenotypic maintenance of a subset of gut-intrinsic γδ T cells is evolutionarily conserved. Although the selected CD103^+^Vγ4^+^ cells often do not dominate the yS compartment, which is in contrast to mice, the cells’ phenotype is unique. Moreover, persons homozygous for hypomorphic BTNL8*3 displayed significant reductions in Vγ4^+^ cell frequencies and the residual cells were phenotypically altered, both of which phenotypes closely phenocopy the situation in mice with deficiencies in BTNL1 or BTNL6 (3, 4). In the settings of BTNL deficiency, the incomplete loss of Vγ4^+^ cells in humans and of Vγ7^+^ cells in mice is likely attributable to the potential of other interactions, e.g., clonotypic antigen reactivities, to promote the amplification and survival of some human Vγ4^+^ and mouseVγ7^+^ T cells, respectively.

The combined loss and phenotypic perturbation of CD103^+^Vγ4^+^ cells was also the single most overt yS cell dysregulation in inflamed IBD. Whereas this dysregulation may in part be a consequence of pathology (i.e., it is a disease biomarker), causative contributions could be surmised from the association of CD severity with homozygosity for BTNL3/8 hypomorphism. Because UC currently does not benefit from the same objectively defined disease behavioural phenotypes, we could not subject it to the same analysis. Nonetheless, throughout our study patients with UC and CD showed conspicuously similar γδ cell dysregulation, and patients with either disease who displayed a renormalised CD103^+^γδ cell compartment showed significantly higher post-treatment remission rates than those with less obvious renormalisation, when assessed five years later. Limitations of this part of our study include the cohort size and the lack of paired longitudinal samples. Thus, we hope that a larger study will now be undertaken, since validation of our findings would pertain to two key points.

First, that routine mucosal CD103^+^Vγ4^+^ cell status assessment may offer a practical prognostic indicator. Whereas histological remission in UC is currently offering improved indicators of disease outcome, 50% of patients in the recent ERIca study who showed histological remission progressed to relapse, highlighting their ongoing, undetected dysregulation (54). Thus, a focus on local γδ T cell dynamics may offer disease-stage specific molecular signatures akin to those proving beneficial in managing bowel cancers (55).

The second key point is the probability that CD103^+^Vγ4^+^ cell renormalisation is likely to be of physiologic benefit. While the nature of that benefit remains to be elucidated, the cells’ wound-healing and anti-inflammatory potentials seem evident in their expression of several potentially relevant genes including *API5* (11), and *FGF9* (24). A capacity of CD103^+^Vγ4^+^ cells to promote epithelial repair would be another conserved feature, given that the exacerbated pathology in γδ-deficient mice experiencing chemically induced colitis is ascribed to defective wound healing (9). From this perspective, the lack of significant benefit versus placebo observed during Phase 3 UC maintenance trials of etrolizumab, that targets CD103, might in part reflect its antagonising homeostatic CD103^+^Vγ4^+^cells (56, 57). Irrespective of the basis of their wound-healing potential, our study has shown that BTNL-selected CD103^+^Vγ4^+^ cells are largely unique in terms of their sensory machinery permitting them to patrol the epithelium (58). That machinery is composed of the TCR; FcsRIy that can mediate signalling from the TCR and from NK receptors; and several NK receptors, particularly NKp46 that can recognise cells undergoing endoplasmic reticulum (ER) stress which is a feature of the colonic epithelium in IBD (59).

It is formally the case that BTNL3/8 loss-of-function might influence IBD progression via a mechanism independent of CD103^+^Vγ4^+^ cell regulation. Nonetheless, the only clearly established biochemical properties of BTNL3+8 heteromers, and of their mouse counterparts, is to engage intestinal γδ TCR Vγ chains. Interestingly, direct evidence germane to the importance of CD103^+^Vγ4^+^ cell regulation may in the near future be provided by analyses of IBD incidence and progression in individuals carrying recently described *TRGV4* alleles encoding chains that show reduced responsiveness to BTNL3+BTNL8 (60).

In this regard, a further two points merit clarification. First, because Vγ4^+^ cells appear highly sensitive to BTNL3+BTNL8 engagement, the reduced levels of BTNL3+BTNL8 expression reported for IBD (15, 46) are not necessarily causal to Vγ4^+^ cell dysregulation, by contrast to either BTNL loss-of-function in donors homozygous for BTNL8*3 or the IBD-associated cytokine milieu. Indeed, upstream disease-associated factors contributing to Vγ4^+^ cell dysregulation would seem consistent with the cells being more germane to disease progression than to disease incidence. Second, it was reported that whereas decreased BTNL3 and ablated BTNL8 expression in celiac disease could renormalise, this did not correlate with renormalisation of either intestinal Vγ1^+^ cells or *TRGV4* reads (14), which is also consistent with other factors contributing to the cells’ dysregulation. That we have observed renormalisation in a cohort of patients with treated IBD may simply reflect disease-specific and treatment-specific factors distinguishing IBD from celiac disease, including but not limited to the fact that celiac disease is largely associated with increases in γδ T cell frequencies whereas we show IBD to be associated with γδ T cell depletions.

Finally, we note that BTNL is not a hit in GWAS for disease susceptibility in either publicly available or our own datasets. BTNL variants have also not previously shown association with disease progression but neither the imputation of our variant of interest nor clinical classifications used in previous international meta-analyses can be assumed to be sufficiently reliable to assess this. By comparison to ~300 loci associated with IBD susceptibility, only few polymorphisms disposing to IBD prognosis have been identified; specifically, *FOXO3, XACT, IGFBP1*, and *HLA*, which were identified via GWAS of patients with indolent and clinically more severe CD (61), rather than stricturing versus penetrating disease behaviours used here. While the mechanistic impact of the few progression-associated genes has yet to be fully elucidated, such genes and pathways may offer useful prognostic indicators by comparison to the arguably disappointing performance of genes disposing to susceptibility (62, 63). Hence, the identification of the BTNL-γδ axis as a disease modifier may offer new insights into understanding and tracking IBD progression. Furthermore, improved tissue repair, by contrast to anti-inflammatory agents, might achieve long-term, drug-free disease remission, which is of particular importance given that IBD is common and is an increasing public health issue in more recently industrialised countries where prevalence continues to rise (64, 65).

## Materials and Methods

### Human Samples

#### Human colonic samples

Up to 12 endoscopic biopsies were obtained from the colon of adult donors undergoing diagnostic colonoscopy after informed consent and in compliance with local ethical approval (REC number 07/H0803/237 or 16/LO/0642). The location and macroscopic appearances of the biopsy site were carefully noted.

Non-IBD controls (Ctrl) were defined as persons undergoing colonoscopy for non-IBD indications where no inflammatory intestinal disease was found and when either none or minimal distant pathology, e.g., minor diverticulosis or sporadic adenomas, were found. In patients who underwent colonoscopy to investigate chronic diarrhoea, histology was taken to exclude microscopic or collagenous colitis. Patients with coeliac disease or other significant intestinal co-morbidity were excluded.

Donors with inflammatory bowel disease (IBD) were identified as either patients with a known history, or symptoms suggestive of IBD prior to endoscopy and prospectively consented. Sampling was performed either in macroscopically uninflamed regions (IBD, Crohn’s disease uninflamed (CD) or ulcerative colitis uninflamed (UC)) or macroscopically inflamed regions (IBDI, CDI or UCI). Demographic details are provided (table S1).

Remission was defined according to strict criteria. Endoscopic remission was defined using validated endoscopic scores: Ulcerative Colitis Endoscopic Index of Severity ≤1 and Simple Endoscopic Score for Crohn’s Disease <3 (53) or Rutgeert’s score i0-1 (66). Patients were also in clinical remission, with no evidence of ongoing biochemical or radiological inflammation. Relapse was defined as symptomatic, endoscopic, radiological, or biochemical relapse leading to treatment escalation, surgery or admission.

Samples obtained at endoscopy were placed directly into Hanks balanced salt solution (HBSS). Up to 4h post collection they were placed in cell culture or frozen in 90% FCS and 10% DMSO.

#### Blood samples

Blood samples were obtained from cone blood from the NHS Blood and Transplant service (London, UK). Peripheral blood mononuclear cells (PBMC) were isolated using the Ficoll-Paque method and frozen in 10% DMSO 50% FCS RPMI.

This study was conducted adhering to the principles of the Declaration of Helsinki.

### DNA and RNA isolation from whole tissue

A single colonic biopsy was placed in RNAlater (ThermoFisher) at the time of acquisition. AllPrep DNA/RNA Mini kit (Qiagen) was used to extract mRNA and genomic DNA from donor tissue following manufacturer’s instructions. Qiagen TissueLyser II was used to homogenise tissue samples.

### TCR deep sequencing

Total RNA extracted from colonic biopsies was sent for γδ TCR chain *(TRDV/TRGV)* deep-sequencing using the IlluminaMiSeq platform with short-read 100/150 PER primers (iRepertoire, USA). Shannon’s entropy and D50 were calculated as previously described (67).

### qPCR

cDNA was synthesized using Superscript-II (Invitrogen) according to manufacturer’s instructions. Gene expression was analysed using the PowerUp SYBR Green Master Mix (Invitrogen) following manufacturer’s instructions. Reactions were run on ViiA7 Real-time PCR machine (Applied Biosystems). Target gene expression was normalised to *RSP9* expression using the 2^-ACT^ method. *RPS9* primer sequences were from Bamias et al (68). *TNF* primer sequences were from Rajput et al (69).

L3qPCR_Ex4F 5' - TGTTGTCATGGGGATGATAATTG-3’
L3qPCR_Ex8R 5'- CATACCACCCTACATTTTGTCCC-3’
L8qPCR_Ex4F 5'- GGCATTGTTGGACTGAAGATTTTC-3’
L8qPCR_Ex8R 5'- CGCCACCTTTTATTGTGTCCTC-3’
RSP9 _F 5' - TCCTTTTCCACTTCGTCTGAG-3’
RSP9_R 5' - AAGCTGATCGGCGAGTATG-3’
TNF_F 5' -ATGAGCACTGAAAGCATGATCC-3’
TNF_R 5' -GAGGGCTGATTAGAGAGAGGTC-3’
Other primers:
Quantitect IL-12A primers (Qiagen #QT00000357)
Quantitext IL-18 primers (Qiagen #QT00014560)
Quantitect IL-23A primers (Qiagen #QT00204078)

### Intestinal lymphocyte isolation

#### Explant method

Human colonic lymphocytes were isolated as previously described (3) and used after a short-term crawl out (36-48h) without the addition of exogenous cytokines, or after 5-7 days of culture in the presence of cytokines when cells were used in co-culture assays or cytokine skewing experiments (see below).

#### Digestion

Whole colonic biopsies were digested using Miltenyi’s Tumour Dissociation Kit (Miltenyi Biotec) according to manufacturer’s instructions for the isolation of lymphocytes.

### Primary cell culture conditions

Samples used in co-culture assays and cytokine skewing experiments were isolated using the explant method and cultured for 5 days in complete media supplemented with IL2 (100 U/mL, Novartis Pharmaceutical UK) and IL15 (10 ng/mL, Biolegend). 1ml of medium was aspirated every second day and replaced with complete medium containing 2x concentrated cytokines. In cytokine skewing experiments, media was also supplemented with IL2 (100 U/mL), IL15 (10 ng/mL), IL1β (10 ng/mL, Peprotech) and IL18 (50 ng/mL, Medical and Biological Laboratories); or with IL2 (100 U/mL), IL15 (10 ng/mL), IL1β (10 ng/mL Biolegend), IL23 (10 ng/mL, Biolegend); or with IL2 (100 U/mL), IL15 (10 ng/mL), TNFα (50 ng/ml, Biolgened).

### Flow cytometry

Flow cytometry was performed using the following antibodies, coupled to the indicated fluorochromes (clones indicated in brackets). Antibodies were purchased from Biolegend unless otherwise stated in brackets. Viability dye Aqua was from Invitrogen. Viability dye Zombie NIR was from Biolegend. Ultra-LEAF™ Purified Human IgG_1_ Isotype Control antibody (QA16A12) was from Biolegend. The following antibodies were used: V S2-AF700 (B6); Vγ2-PerCpCy5.5 (B6); ySTCR-PE.Cy7 (IMMU510; Beckman Coulter); Vγ1-FITC (TS8.2; ThermoFisher); Vγ1-PE (REA173; Miltenyi); Vγ1-APC (REA173; Miltenyi); CD3-BV786 (OKT3); CD3-BV510 (OKT3); CD8α-BV510 (HIT8a); CD8a-PerCpCy5.5 (HIT8a); CD8α-FITC (HIT8a); CD103-FITC (Ber-ACT8); CD103-BV605 (Ber-ACT8); CD103- PerCpCy5.5 (Ber-ACT8); CD103-BV421 (Ber-ACT8); CD5-BV421 (L17F12); CD18-PE (1B4/18); 41BB-BV605 (4B4-1); IFNγ-BV421 (4S.B3); TNFα-APC (MAb11); IL17A- BV605 (BL168); CD101-PerCpCy5.5 (BB27); TIGIT-PerCpCy5.5 (A15153D); TIGIT-PerCpCy5.5 (741182; BD Biosciences); Nkp30-BV711 (P30-15); Nkp44-BV605 (p44-8); Nkp46-PE (9E2); Nkp46-BV421 (9E2); CD49a-APC (TS2/7); CD69-BV510 (FN50); CD38- BV605 (HB-7); ICOS-BV711 (C398.4A); NKG2C-BV421 (134591; BD Biosciences); CCR9-APC (L053E8); CD31-BV421 (WM59); CD96-BV421 (NK92.39); NKG2D-BV605 (1D11); DNAM1-BV605 (DX11; BD Biosciences); CCR7-APC (G043H7); CCR7-BV421(G043H7); NKG2A-BV711 (131411; BD Biosciences); TIM3-BV421 (F38-2E2); FcsRIy-FITC (FCABS400F; Merck); CD107a-PE (LAMP1); IL2-PE (MQ1-17H12); CD45RA-BV421 (HI100); CD27-BV786 (O323). The biotin-conjugated antibody against Vγ2/3/4 (23D12) (70) was detected by conjugation to BV650 or PE-streptavidin (Biolegend). The anti-Vγ4 antibodies (G4_9 and G4_18; Iontas) were human IgG_1_ isotype and goat anti-human IgG (H+L)-AF647 (polyclonal; ThermoFisher) was used to detect unconjugated clones. In addition, clone G4_18 was conjugated to AF647 using the Lightning Link Fast conjugation kit (Abcam) following manufacturer’s instruction with the exception that the incubation period with modifier agent was extended to overnight at 4°C. Primary cells were fixed with BD CellFIX (BD Biosciences) for 20 min at room temperature prior to acquisition.

For cytokine stains, lymphocytes were stimulated with 10 ng/ml PMA (phorbol 12-myristate 13-acetate - Merck) and 1 μg/ml ionomycin (Merck) in the presence of 20 μg/ml BFA (Merck) for 4 h at 37°C 5% CO_2_. Following surface staining and cell fix, lymphocytes were permeabilized with Intracellular Staining Permeabilization Wash Buffer (Biolegend) for 20 min at room temperature. The relevant conjugated monoclonal antibodies were added to the permeabilization buffer to the cells and incubated for another 20 min. The cells were washed and acquired.

When examining degranulation in the co-culture assay, conjugated anti-CD107a-PE antibody was added at the start of a 6 h co-culture assay and surface staining was performed at the end of the assay.

Experiments were acquired on BD LSRFortessa X-20 or BD FACSCanto II. Flow cytometry data analysis was performed on FlowJo (Version 10).

### Generation of anti-human Vγ4 antibodies

Anti-Vγ4 TCR antibodies were generated by Iontas and provided by Adaptate Biotherapeutics. They were derived using phage display following industry standard techniques (71). A discovery strategy was devised to exert selection pressure towards the Vγ4Vγ1 and Vγ4Vγ2 TCR complexes. To this end, phage display selections were performed with recombinant TCR antigens in solution phase. Binders were screened in soluble scFv format by subcloning the entire selection outputs in a suitable scFv expression vector. Hits were assessed by specific binding to Vγ4Vγ1 and Vγ4Vγ2 TCR antigens and not Vγ2Vγ1, Vγ2Vγ2, Vγ8Vγ1 TCR antigens. Unique binder sequences were ranked according to their relative affinity to Vγ4 in a scFv capture ELISA. A final selection of 24 binders with lowsequence liability, varying estimated affinities and CDR3 diversity were chosen for IgG conversion. Of the 24 candidates, 2 were validated to bind Vγ4^+^ TCR specifically, with good staining indexes and with minimal background signal (fig. S3A).

### Cell lines

HEK293T cells were from the ATCC and were maintained in DMEM (supplemented with 10% FBS and 100 U/mL Penicillin-Streptomycin). Human TCRβ^-^ cell line JRT3 was a gift from Dr Salah Mansour, University of Southampton and was cultured in RPMI 1640 (supplemented with 10% FBS and 100 U/mL Penicillin-Streptomycin).

Cells transduced with empty vector (EV) or BTNL3 and BTNL8 have previously been described (3) and were cultured in DMEM (10% FBS, 100 U/mL Penicillin-Streptomycin) supplemented with 1 μg/mL puromycin (Sigma-Aldrich) and 500 μg/mL hygromycin (Thermo Fisher).

### Plasmids, cloning, transfection and lentiviral transduction

Plasmids encoding hu17 (Vγ4Vγ1), hu17.Vγ2 (Vγ2Vγ1), hu17.Vγ3 (Vγ3Vγ1), hu17.Vγ3-Vγ4^HV4^(Vγ3Vγ1), hu17.Vγ3-Vγ4^CDR2-HV4^(Vγ3Vγ1), hu20 (Vγ4Vγ1) and huPB (Vγ9Vγ2) have previously been described (12, 13). The plasmid encoding hu20/huPB (Vγ4Vγ2) was generated by subcloning the hu20 gamma chain using NcoI/XbaI and the huPB delta chain using XhoI/NotI into the self-inactivating lentiviral vector pCSIGPW after removal of the IRES-GFP and CMVp-Puro^R^ cassettes (3, 48).

The self-inactivating lentiviral vector pCSIGPW (SFFV promoter – Multiple Cloning Site [MCS] – IRES-GFP – CMV promoter – Puromycin^R^), and plasmids encoding FLAG-BTNL3 and FLAG-BTNL8 were previously described (3). The FLAG-BTNL8*3 construct was derived from FLAG-BTNL8 and FLAG-BTNL3 plasmids by using a unique DraIII restriction site at the beginning of the B30.2 domains of BTNL8 and BTNL3.

All plasmids were transduced and transfected following a previously established protocol (12, 13).

### Co-culture assays

5x10^5^ HEK293T cells, transduced with either EV (293T.EV), BTNL3 and BTNL8 (293T.L3L8) or BTNL8 and BTNL8*3 (293T.L8L8*3) were co-cultured with 2x10^5^ primary human lymphocytes harvested after 5 days of culture in the presence of IL2 and IL15 as previously described (12). Cells were co-cultured overnight at 37°C, 5% CO_2_ in 96-well round bottom plates with complete medium without supplementary cytokines. Cells were subsequently analysed by flow cytometry.

### RNA sequencing

#### RNA sequencing of human intestinal CD103^+/neg^ Vγ2/3/4^+^ cells

Intestinal lymphocytes were isolated using the Miltenyi tumour dissociation kit as described above. Cells were sorted directly into TRIzol n=1 or PBS n=2 and sent to GENEWIZ (Bishop’s Stortford, UK) for RNA extraction, cDNA was prepared with an ultralow input. Single-end 150 bp sequencing was undertaken on an Illumina Hiseq. The final sample (Sample A) was FACS sorted directly into lysis buffer and RNA isolated via the PicoPure kit (Thermofisher) as per manufacturer’s instructions. cDNA was prepared with Ovation RNA-Seq System V2 (NuGEN) and libraries were constructed with Ultralow Library System V2 (NuGEN) and sequenced single-end sequence read 75 bp undertaken on an Illumina Hiseq 2500. Single-end reads were aligned to human genome GRCh38 from Ensembl using STAR (v2.5.2a). Gene-level quantifications using annotation release 86 from Ensembl were generated using RSEM v1.3.0. To identify differential expression raw gene level counts were imported into DESeq2 (72). Genes with a mean normalized count below zero across all samples were removed prior to statistical analysis.

The data have been deposited in NCBI's Gene Expression Omnibus (GEO) under accession number GSE218058 (https://www.ncbi.nlm.nih.gov/geo/query/acc.cgi?acc=GSE218058) (73).

#### RNA sequencing of human intestinal Vγ2/3/4^+^ cells co-cultured with 293T.EV or 293T.L3L8 cells.

Intestinal lymphocytes were isolated using the explant grid culture method for two weeks in IL2 and IL15. Cells were then harvested and co-cultured overnight with 293T.EV or 293T.L3L8. Vγ2/3/4^+^ cells from each condition were sorted directly into lysis buffer and RNA was extracted using the RNEasy MicroPlus kit (Qiagen) following manufacturer’s instructions. RNA was sent for library preparation and single-end sequencing on Illumina HiSeq 2500 at the Advanced Sequencing Facility (The Francis Crick Institute).

75 base-pair single-end reads were aligned to human genome GRCh38 from Ensembl using STAR (v2.5.2a). Gene-level quantifications using annotation release 86 from Ensembl were generated using RSEM v1.3.0. To identify differential expression raw gene level counts were imported into DESeq2(72). Genes with a mean normalized count below zero across all samples were removed prior to statistical analysis. Differential expression between the Vγ2/3/4^+^ cells co-cultured with 293T.EV and 293T.L3L8L3+L8 groups was identified by taking into account the paired structure within the replicate groups. Using an FDR of 0.01 37 culture-condition-dependent gene expression effects were identified.

The data have been deposited in NCBI's GEO under the accession number GSE224412 (https://www.ncbi.nlm.nih.gov/geo/query/acc.cgi?acc=GSE224412) (73).

#### RNA sequencing of human intestinal γδ T cells

11 biopsies from control donors were digested and live Vγ4^+^Vγ2/3/4^+^γδTCR^+^CD3^+^Vγ2^neg^ and Vγ4^neg^Vγ234^neg^γδTCR^+^CD3^+^Vγ2^neg^ cells were sorted directly into extraction buffer from the PicoPure RNA Isolation Kit (ThermoFisher). RNA was isolated immediately, adhering to manufacturer’s instruction. Total RNA was stored at -80°C freezer until submission. Libraries were prepared by the Advanced Sequencing facility at the Francis Crick Institute using the NEBNext Ultra II RNA Library prep kit. Libraries were sequenced by the BRC Genomics Facility at Guy’s hospital on Illumina NextSeq 2000.

All software for the data analysis was run with default settings, unless otherwise indicated. The quality of the sequencing reads was examined using FastQC (v0.11.4). Raw sequencing reads (100-nt, paired-end) were trimmed using Trimgalore (v0.4.4). Traces of ribosomal DNA and mitochondrial DNA were removed using the Bowtie2 (v2.2.5) (74). Reads were aligned to the human reference genome GRCh38 using STAR (v2.5.3a) with default setting (75). Duplicate reads were removed using the MarkDuplicates function of the Picard tools (v2.17.11). Reads were annotated using the Partek E/M (Partek software) gene count. Differentially expressed genes (DEG) were identified using DESeq2 (v3.5) (72). All software for the data analysis was run with default settings, unless otherwise indicated. The quality of the sequencing reads was examined using FastQC (v0.11.4). Raw sequencing reads (100-nt,paired-end) were trimmed using Trimgalore (v0.4.4). Traces of ribosomal DNA and mitochondrial DNA were removed using the Bowtie2 (v2.2.5) (74). Reads were aligned to the human reference genome GRCh38 using STAR (v2.5.3a) with default setting (75). Duplicate reads were removed using the MarkDuplicates function of the Picard tools (v2.17.11). Reads were annotated using the Partek E/M (Partek software) gene count. Differentially expressed genes (DEG) were identified using DESeq2 (v3.5) (72).

The data have been deposited in NCBI's Gene Expression Omnibus (GEO) and are accessible through GEO Series accession number GSE218446 and at the following link: https://www.ncbi.nlm.nih.gov/geo/query/acc.cgi?acc=GSE218446 (73).

#### Gene Set Enrichment Analysis (GSEA)

GSEA pre-ranked analysis was performed using DEGs between Vγ2/3/4 CD103^+^ (positive values) and CD103^neg^ (negative values) with several published datasets (Fig. 1D, E; fig. S1E) (76).

For human datasets the published gene signature of the cells under investigation was used. To compare the published murine Vγ7^+^ data, murine genes were converted to human orthologues using https://biit.cs.ut.ee/gprofiler/gost (77). Three published gene signatures were large (3, 25, 26), hence the top 500 significantly DEG were taken as the gene signature.

### DNA samples for genotyping experiments

The study included 12,767 unrelated UK patients with IBD. The ethnicity of both cases and controls was limited to White European ancestry as these alleles are underrepresented in other ethnicities (47). The study included a test set of 4517 IBD patients consisting of patients recruited after ethical review and obtaining of informed consent from Guy’s and St. Thomas’ Hospitals London, United Kingdom and the Royal Victoria Infirmary, Newcastle, United Kingdom (REC 12/YH/0172). The replication set included 8250 IBD patients incorporated into the NIHR IBD bioresource. The diagnosis of IBD was made by established criteria of clinical, radiologic, and endoscopic analysis and from histology reports. Patients were classified according to the Montreal Classification into disease location (L1-3) and disease behavior (B1-3). Duration of disease was defined as onset of symptoms or date of diagnosis to date of inclusion in the database. B1 cases were included only if they had at least 5 years documented follow up to avoid incorporating patients who had not yet progressed to other phenotypes. B2 and B3 disease were recorded regardless of disease duration. Only patients with CD and sufficient phenotypic and disease duration data were included in the final analysis of genetic association with disease behaviour (test n=1817, replication n=4491).

The study also included 6767 population controls, obtained from the 1958 British Birth Cohort, including all subjects born between 3^rd^ and 9^th^ March 1958 in England, Scotland, and Wales (National Child Development Study: http://www.cls.ioe.ac.uk) (49).

### BTNL8*3 CNV genotyping by PCR

All locally sourced gDNA obtained from donors was used at 10 ng/reaction. PCR was performed using Q5 High Fidelity DNA polymerase (NEB) following manufacturer’s instructions and in a single tube reaction. The following primers were used:

L8_F 5’-GACTTTTCACCCCCAAAACC-3’
L8_R 5’-CTCATGTCCACCTGAGATTCTG-3’
L3_R 5’-GTAAGCCCTCTATTTGACTTTGTG-3’

Predicted product sizes were 1602 bp for BTNL8*3 and 1310 bp for BTNL8. PCR product was separated by 1% agarose gel.

### Identification of rs72494581 as a surrogate SNP of the BTNL8*3 CNV

We cloned and sequenced the PCR products generated by amplification of the genomic region resulting from the CNV using primers L8_F and L3_R (sequences above), which generates a 1602 bp product (fig. S6A). After aligning the obtained sequences from 6 different CNV homozygous donors (GUT1-6) to the reference sequences of the genomic regions of BTNL8 (L8) and BTNL3 (L3), as well as the predicted sequence of the CNV allele (L8*3), we confirmed the location of the non-allelic homologous recombination region suggested by (47) (fig. S6B). Of note, this region starts with a sequence that is near-identical to a recombination hot spot sequence reported by Myers et al. (78).

The CNV alleles from all 6 donors all included a T>C SNP (rs72494581, not reported by Aigner et al (47)). We then compared the results of the RT-PCR method using L8_F, L8_R and L3_R primers (sequences above and fig S6A) using gDNA from donors that had previously been genotyped with a custom TaqMan-based genotyping assay for rs72494581 (fig. S6C). Given the relatively limited number of samples tested, we cannot guarantee that rs72494581 is in complete LD in all cases but given its proximity to the putative break point (<200bp) and the perfect match between the RT-PCR and TaqMan data (fig S6A, C), this SNP is potentially the best marker of the CNV allele.

### BTNL8*3 CNV genotyping by TaqMan assay

Custom TaqMan SNP genotyping assay for rs72494581 was ordered from ThermoFisher and used as per manufacturer’s instructions with TaqMan Genotyping Master Mix (ThermoFisher) and a minimum of 4 ng of each gDNA sample per reaction. Post-amplification allelic discrimination was carried out using the ABI7900HT (TaqMan plate reader, end-point read). Case control association test was performed by Chi-squared test. Primer sequences were:

rs72494581_F 5' -CTCGTCCCACCTGTGCAA-3’
rs72494581_R 5'- GAAACAGTATATTCAGTGGTGGACACA-3’

### KASP genotyping

A KASP assay for rs72494581 was custom designed and validated against known samples. High throughput genotyping was outsourced and undertaken by KASP genotyping (LGC Biosearch Technologies, Hoddesdon, UK). Clinical data relating to patients tested for rs72494581 was provided by the NIHR IBD Bioresource.

### Statistical analysis

With the exception of sequencing analysis (see above), all other statistics were performed in GraphPad Prism v9. Statistical information is included in the figure legends. In all figures horizontal bars denote the median and error bars denote the minimum and maximum datapoints.

## Supplementary Material

Data S1

MDAR reproducibility checklist

Supplementary figures

Table S1

Table S2

Table S3

Table S4

## Data Availability

All data are available in the manuscript or the supplementary materials or are deposited as follows: sequencing data generated for this study is available on NCBI GeneExpression Omnibus (GEO) under accession numbers GSE218058, GSE224412 and GSE218446. All reagents described in this paper derived for these studies will be shared upon request with other researchers in academia. Any MTAs will be organised upon receipt of request to the corresponding authors.

## One-Sentence Summary

A unique human gut γδ T cell subset, the perturbation of which is a risk factor for poor inflammatory bowel disease outcome.

## References

[1] Fernando MMA (2008). Defining the Role of the MHC in Autoimmunity: A Review and Pooled Analysis. PLOS Genetics.

[2] Hayday AC (2022). Paul’s Fundamental Immunology.

[3] Di Marco Barros R (2016). Epithelia Use Butyrophilin-like Molecules to Shape Organ-Specific gammadelta T Cell Compartments. Cell.

[4] Jandke A (2020). Butyrophilin-like proteins display combinatorial diversity in selecting and maintaining signature intraepithelial gammadelta T cell compartments. Nature communications.

[5] Barbee SD (2011). Skint-1 is a highly specific, unique selecting component for epidermal T cells. Proceedings of the National Academy of Sciences.

[6] Girardi M (2002). Resident skin-specific gammadelta T cells provide local, nonredundant regulation of cutaneous inflammation. The Journal of experimental medicine.

[7] Muñoz-Ruiz M (2022). Tissue-intrinsic γδ T cells critically regulate Tissue-Resident Memory CD8 T cells. bioRxiv.

[8] Kuhl AA (2007). Aggravation of intestinal inflammation by depletion/deficiency of gammadelta T cells in different types of IBD animal models. Journal of leukocyte biology.

[9] Chen Y, Chou K, Fuchs E, Havran WLR (2002). Boismenu, Protection of the intestinal mucosa by intraepithelial gamma delta T cells. Proceedings of the National Academy of Sciences.

[10] Inagaki-Ohara K (2004). Mucosal T cells bearing TCRgammadelta play a protective role in intestinal inflammation. Journal of immunology.

[11] Matsuzawa-Ishimoto Y (2022). The gammadelta IEL effector API5 masks genetic susceptibility to Paneth cell death. Nature.

[12] Melandri D (2018). The γδ T cell receptor combines innate with adaptive immunity by utilizing spatially distinct regions for agonist-selection and antigen responsiveness. Nature immunology.

[13] Willcox CR (2019). Butyrophilin-like 3 Directly Binds a Human Vgamma4(+) T Cell Receptor Using a Modality Distinct from Clonally-Restricted Antigen. Immunity.

[14] Mayassi T (2019). Chronic Inflammation Permanently Reshapes Tissue-Resident Immunity in Celiac Disease. Cell.

[15] Lebrero-Fernandez C (2016). Altered expression of Butyrophilin (BTN) and BTN-like (BTNL) genes in intestinal inflammation and colon cancer. Immun Inflamm Dis.

[16] Cerf-Bensussan N (1987). A monoclonal antibody (HML-1) defining a novel membrane molecule present on human intestinal lymphocytes. European journal of immunology.

[17] Cepek KL (1994). Adhesion between epithelial cells and T lymphocytes mediated by E-cadherin and the alpha E beta 7 integrin. Nature.

[18] Cantoni C (1998). p49, a putative HLA class I-specific inhibitory NK receptor belonging to the immunoglobulin superfamily. European journal of immunology.

[19] Wermers JD, McNamee EN, Wurbel MA, Jedlicka P, Rivera-Nieves J (2011). The chemokine receptor CCR9 is required for the T-cell-mediated regulation of chronic ileitis in mice. Gastroenterology.

[20] Voskoboinik I, Whisstock JC, Trapani JA (2015). Perforin and granzymes: function, dysfunction and human pathology. Nature Reviews Immunology.

[21] Okamura H, Kashiwamura S, Tsutsui H, Yoshimoto T, Nakanishi K (1998). Regulation of interferon-gamma production by IL-12 and IL-18. Curr Opin Immunol.

[22] Deaglio S (2007). Adenosine generation catalyzed by CD39 and CD73 expressed on regulatory T cells mediates immune suppression. Journal of Experimental Medicine.

[23] Monney L (2002). Th1-specific cell surface protein Tim-3 regulates macrophage activation and severity of an autoimmune disease. Nature.

[24] Gay D (2013). Fgf9 from dermal gammadelta T cells induces hair follicle neogenesis after wounding. Nature medicine.

[25] Atlasy N (2022). Single cell transcriptomic analysis of the immune cell compartment in the human small intestine and in Celiac disease. Nature communications.

[26] Kumar BV (2017). Human Tissue-Resident Memory T Cells Are Defined by Core Transcriptional and Functional Signatures in Lymphoid and Mucosal Sites. Cell Rep.

[27] Fitz Patrick MEB (2021). Human intestinal tissue-resident memory T cells comprise transcriptionally and functionally distinct subsets. Cell Rep.

[28] Sallusto F, Lenig D, Forster R, Lipp MA (1999). Lanzavecchia, Two subsets of memory T lymphocytes with distinct homing potentials and effector functions. Nature.

[29] Blackford J, Reid HW, Pappin DJ, Bowers FS, Wilkinson JM (1996). A monoclonal antibody, 3/22, to rabbit CD11c which induces homotypic T cell aggregation: evidence that ICAM-1 is a ligand for CD11c/CD18. European journal of immunology.

[30] Azzam HS (1998). CD5 expression is developmentally regulated by T cell receptor (TCR) signals and TCR avidity. The Journal of experimental medicine.

[31] Orta-Mascaro M (2016). CD6 modulates thymocyte selection and peripheral T cell homeostasis. The Journal of experimental medicine.

[32] Kuo CT, Veselits MLJM (1997). Leiden, LKLF: A transcriptional regulator of single-positive T cell quiescence and survival. Science (New York, NY).

[33] Malhotra N (2013). A network of high-mobility group box transcription factors programs innate interleukin-17 production. Immunity.

[34] Jonsson AH (2022). Granzyme K(+) CD8 T cells form a core population in inflamed human tissue. Science translational medicine.

[35] Groh V (2001). Costimulation of CD8alphabeta T cells by NKG2D via engagement by MIC induced on virus-infected cells. Nature immunology.

[36] McKenzie DR (2022). Normality sensing licenses local T cells for innate-like tissue surveillance. Nature immunology.

[37] Rodewald HR, Arulanandam AR, Koyasu S, Reinherz EL (1991). The high affinity Fc epsilon receptor gamma subunit (Fc epsilon RI gamma) facilitates T cell receptor expression and antigen/major histocompatibility complex-driven signaling in the absence of CD3 zeta and CD3 eta. J Biol Chem.

[38] Rodewald HR (1992). A population of early fetal thymocytes expressing Fc gamma RII/III contains precursors of T lymphocytes and natural killer cells. Cell.

[39] Liu CP (1993). Abnormal T cell development in CD3-zeta-/-mutant mice and identification of a novel T cell population in the intestine. The EMBO journal.

[40] Dechanet J (1999). Implication of gammadelta T cells in the human immune response to cytomegalovirus. The Journal of clinical investigation.

[41] Prinz I (2013). Donor Vγ1+ γδ T cells expand after allogeneic hematopoietic stem cell transplantation and show reactivity against CMV-infected cells but not against progressing B-CLL. Exp Hematol Oncol.

[42] Davey MS (2017). Clonal selection in the human Vdelta1 T cell repertoire indicates gammadelta TCR-dependent adaptive immune surveillance. Nature communications.

[43] Rutgeerts P (2005). Infliximab for Induction and Maintenance Therapy for Ulcerative Colitis. New England Journal of Medicine.

[44] Sandborn WJ (2012). Ustekinumab induction and maintenance therapy in refractory Crohn’s disease. The New England journal of medicine.

[45] D'Haens G (2022). Risankizumab as induction therapy for Crohn’s disease: results from the phase 3 Advance and Motivate induction trials. Lancet (London, England).

[46] Parikh K (2019). Colonic epithelial cell diversity in health and inflammatory bowel disease. Nature.

[47] Aigner J (2013). A common 56-kilobase deletion in a primate-specific segmental duplication creates a novel butyrophilin-like protein. BMC genetics.

[48] Vantourout P (2018). Heteromeric interactions regulate butyrophilin (BTN) and BTN-like molecules governing gammadelta T cell biology. Proceedings of the National Academy of Sciences.

[49] Power CJ (2006). Elliott, Cohort profile: 1958 British birth cohort (National Child Development Study). Int J Epidemiol.

[50] Silverberg MS (2005). Toward an integrated clinical, molecular and serological classification of inflammatory bowel disease: report of a Working Party of the 2005 Montreal World Congress of Gastroenterology. Can J Gastroenterol.

[51] Lo B (2018). Changes in Disease Behaviour and Location in Patients With Crohn’s Disease After Seven Years of Follow-Up: A Danish Population-based Inception Cohort. Journal of Crohn’s colitis.

[52] Jameson J (2002). A role for skin gammadelta T cells in wound repair. Science (New York, NY).

[53] Turner D (2021). STRIDE-II: An Update on the Selecting Therapeutic Targets in Inflammatory Bowel Disease (STRIDE) Initiative of the International Organization for the Study of IBD (IOIBD):Determining Therapeutic Goals for Treat-to-Target strategies in IBD. Gastroenterology.

[54] Rath T (2023). Intestinal Barrier Healing Is Superior to Endoscopic and Histologic Remission for Predicting Major Adverse Outcomes in Inflammatory Bowel Disease: The Prospective ERIca Trial. Gastroenterology.

[55] Le DT (2015). PD-1 Blockade in Tumors with Mismatch-Repair Deficiency. The New England journal of medicine.

[56] Vermeire S (2022). Etrolizumab for maintenance therapy in patients with moderately to severely active ulcerative colitis (LAUREL): a randomised, placebo-controlled, double-blind, phase 3 study. The Lancet Gastroenterology Hepatology.

[57] Peyrin-Biroulet L (2022). Etrolizumab as induction and maintenance therapy for ulcerative colitis in patients previously treated with tumour necrosis factor inhibitors (HICKORY): a phase 3, randomised, controlled trial. Lancet Gastroenterol Hepatol.

[58] Hoytema van Konijnenburg DP (2017). Intestinal Epithelial and Intraepithelial T Cell Crosstalk Mediates a Dynamic Response to Infection. Cell.

[59] Kaser A (2008). XBP1 links ER stress to intestinal inflammation and confers genetic risk for human inflammatory bowel disease. Cell.

[60] Corcoran M (2023). Archaic humans have contributed to large-scale variation in modern human T cell receptor genes. Immunity.

[61] Lee JC (2017). Genome-wide association study identifies distinct genetic contributions to prognosis and susceptibility in Crohn’s disease. Nature genetics.

[62] Cleynen I (2016). Inherited determinants of Crohn’s disease and ulcerative colitis phenotypes: a genetic association study. Lancet (London, England).

[63] Colombel JF, Narula NL (2017). Peyrin-Biroulet, Management Strategies to Improve Outcomes of Patients With Inflammatory Bowel Diseases. Gastroenterology.

[64] Jones G-R (2019). IBD prevalence in Lothian, Scotland, derived by capture-recapture methodology. Gut.

[65] Ng SC (2017). Worldwide incidence and prevalence of inflammatory bowel disease in the 21st century: a systematic review of population-based studies. Lancet (London, England).

[66] Rutgeerts P (1990). Predictability of the postoperative course of Crohn’s disease. Gastroenterology.

[67] Joseph M (2022). Global patterns of antigen receptor repertoire disruption across adaptive immune compartments in COVID-19. Proceedings of the National Academy of Sciences of the United States of America.

[68] Bamias G (2013). Comparative study of candidate housekeeping genes for quantification of target gene messenger RNA expression by real-time PCR in patients with inflammatory bowel disease. Inflammatory bowel diseases.

[69] Rajput S, Volk-Draper LD, Ran S (2013). TLR4 Is a Novel Determinant of the Response to Paclitaxel in Breast Cancer. Molecular Cancer Therapeutics.

[70] Kabelitz D (1994). New monoclonal antibody (23D12) recognizing three different V gamma elements of the human gamma delta T cell receptor. 23D12+ cells comprise a major subpopulation of gamma delta T cells in postnatal thymus. Journal of immunology.

[71] Schofield DJ (2007). Application of phage display to high throughput antibody generation and characterization. Genome Biol.

[72] Love MI, Huber WS (2014). Anders, Moderated estimation of fold change and dispersion for RNA-seq data with DESeq2. Genome Biol.

[73] Edgar R, Domrachev MAE (2002). Lash, Gene Expression Omnibus: NCBI gene expression and hybridization array data repository. Nucleic Acids Res.

[74] Langmead B, Salzberg SL (2012). Fast gapped-read alignment with Bowtie 2. Nat Methods.

[75] Dobin A (2013). STAR: ultrafast universal RNA-seq aligner. Bioinformatics.

[76] Subramanian A (2005). Gene set enrichment analysis: a knowledge-based approach for interpreting genome-wide expression profiles. Proceedings of the National Academy of Sciences of the United States of America.

[77] Raudvere U (2019). g:Profiler: a web server for functional enrichment analysis and conversions of gene lists (2019 update). Nucleic Acids Res.

[78] Myers S, Freeman C, Auton A, Donnelly P, McVean G (2008). A common sequence motif associated with recombination hot spots and genome instability in humans. Nature genetics.

