# Supplementary material for: Conserved γδ T cell selection by BTNL proteins limits progression of human inflammatory bowel disease
Source: Science. Author manuscript; Available in PMC 2023 Sep 26. (PMC7615126; doi:10.1126/science.adh0301)
Supplement: Supplementary figures [file EMS188097-supplement-Supplementary_figures.docx]

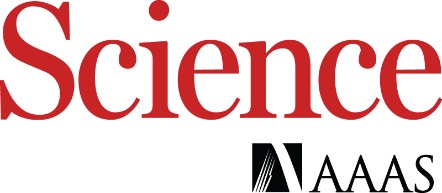


Supplementary Materials for

**Conserved γδ T cell selection by BTNL proteins limits Inflammatory Bowel Disease progression**

**Authors:** Robin J Dart^1,2,3*†^, Iva Zlatareva^1,2†^, Pierre Vantourout^1,2^, Efstathios Theodoridis^1,2^, Ariela Amar^4^, Shichina Kannambath^5^, Philip East^6^, Timothy Recaldin^7^, John C Mansfield^8,9^, Christopher A Lamb^8,9^, Miles Parkes^10^, Peter M Irving^3^, Natalie J Prescott^4^ and Adrian C Hayday^1,2*^

Correspondence to: robin.dart@kcl.ac.uk; adrian.hayday@crick.ac.uk

**The PDF file includes:**

Figs. S1 to S5

Tables S1 to S4

Data S1

**Figure S1.** **The phenotype of human gut γδ T cells. (A).** Representative flow plots of Vδ1 and Vδ2 expression on γδ T cells derived from peripheral blood mononuclear cells (PBMCs) and colon. **(B).** *TRDV* gene usage in control colonic biopsies measured by mRNA TCR deep sequencing (n=4). **(C).** Flow cytometric analysis of percent Vδ2^+^ (of total CD3^+^), Vγ234^+^ (of Vδ2^neg^) and Vδ1^+^ (of Vγ2/3/4^+^) cells in control biopsies (n=14). **(D).** Corelation plot between %*TRGV4* and %*TRGV2* reads from donors shown in Fig. 1A. Spearman correlation. **(E).** Gene Set Enrichment Analysis (GSEA) of the human CD103^+^Vγ2/3/4^+^ signature from Fig. 1D. in a published human intestinal CD8^+^CD103^+^ αβ T cell signature dataset (27)**.** NES – normalized enrichment score. **(F).** Flow cytometric analysis of the expression of the indicated markers (y axis) on CD103^+^ and CD103^neg^ Vγ2/3/4^+^ cells. Representative flow plots (top) and summary data (bottom) are shown (n=3-7). Connecting lines – paired data. Paired *t*-test, **p*<0.05; ****p*<0.001.

**Figure S2.** **Vγ2/3/4^+^ cells make functional responses to BTNL3+BTNL8 stimulation but do not make classical effector cytokine responses.** **(A).** Flow cytometric analysis of CD3 geometric mean fluorescent intensity (gMFI) on intestinal Vγ2/3/4^+^ cells from control donors after overnight co-culture with 293T.EV or 293T.L3L8 (n=18). Paired *t*-test, *****p*<0.0001. **(B).** Representative flow cytometry plots of CD3 expression on Vγ2/3/4^+^ cells from control donors co-cultured as in A. **(C).** Correlation plots of %*TRGV* reads vs %TCR downregulation in response to 293T.L3L8 from the same donors (n=22). Spearman correlation coefficient. **(D).** Gut lymphocytes were co-cultured overnight with 293T.EV or 293T.L3L8 cells and Vγ2/3/4^+^ lymphocytes were subsequently sorted for RNA sequencing (n=4). Gating strategy (left) and summary data of TCR downregulation of sorted donors (right) are shown. **(E).** Flow cytometric analysis of CD107a expression on Vγ2/3/4^+^ cells after 6h co-culture with 293T.EV or 293T.L3L8 or cultured in media only (n=3). Example flow plots and summary graph (far fight) are shown. **(F)**. Example flow cytometry plots of TNFα, IFNγ and IL2 expression (x axis) by Vδ2^neg^ γδ T cells and αβ T cells after overnight stimulation with 293T.EV, 293T.L3L8 or P+I. (**G).** Summary graph of the results shown in F (n=3).

**Figure S3. Vγ4 cells are phenotypically distinct from other γδ cells. (A).** Example flow cytometry plots of JRT3 cells transduced with indicated TCRs and stained with pan-γδTCR antibody (top row) and anti-Vγ4 antibody clones G4_9 (middle row) and G4_12 (bottom row). Hu17 – Vγ4Vδ1 TCR; hu17.Vγ2 – Vγ2Vδ1 TCR; hu17.Vγ3 – Vγ3Vδ1 TCR; hu17.Vγ3-Vγ4^HV4^ – Vγ3Vδ2 TCR with HV4γ region from Vγ4 chain; hu17.Vγ3-Vγ4^CDR2-HV4^ – Vγ3Vδ1 TCR with CDR2-HV4γ chain region from Vγ4; hu20/PB – Vγ4Vδ2; LES – Vγ4Vδ5. **(B).** Example flow cytometry plots of FcεRIγ expression (y axis) in Vγ4^+^ and Vγ4^neg^ (x axis) Vδ2^neg^ cells from control gut tissue (CD103^+^ and CD103^neg^) and PBMCs. **(C).** Flow cytometric analysis of % FcεRIγ^+^ cells in the indicated γδ subsets (x axis) from blood (n=5). **(D-F).** Example flow plots of (D) Nkp46, (E) NKG2C and (F) CD31 expression (y axis) in Vγ4^+^ and Vγ4^neg^ (x axis) Vδ2^neg^ cells from control gut tissue (CD103^+^ and CD103^neg^). **(G).** Flow cytometric analysis of % CD38^+^ cells in (left) and CD38 gMFI (geometric mean fluorescent intensity) on (right) the indicated γδ subsets (x axis) from control gut tissue (CD103^+^ and CD103^neg^) (n=4-5). Two-way ANOVA with Dunnett’s correction against Vγ4^+^ cells was used for analysis, * *p*<0.05; *** *p*<0.001.

**Figure S4. Phenotypic and clonotypic changes of the intestinal γδ T cells compartment in IBD.** **(A).** Flow cytometric analysis of Vδ2^+^ cells of total CD3 cells isolated from control (n=34), CD (n=23), CDI (n=18), UC (n=19), UCI (n=14) biopsies. Kruskal-Wallis test with Dunn’s correction against control donors was used for analysis, **p*<0.05. **(B).** Total γδTCR (left) and *TRGV2/3/4* (right) diversity measured by D50 derived from TCR deep sequencing of whole biopsies mRNA from Ctrl (n=13), IBD (n=9) and IBDI (n=9) donors. Open circled – CD donors; filled circles – UC donors. Kruskal-Wallis test with Dunn’s correction (left) and 2-way ANOVA with Dunnett’s correction (right) against control donors was used for analysis, **p*<0.05; ***p*<0.01; ****p*<0.001. **(C).** Flow cytometric analysis of the proportion of CD103^+^CD45RA^+^CD27^+^ and CD103^neg^CD45RA^+^CD27^+^ of Vδ2^neg^ gut cells from control (n=11), IBDU (n=10) and IBDI (n=1) donors. Open circles – CD donors; filled circles – UC donors. Two-way ANOVA with Dunnett correction against control donors was used for analysis, ***p*<0.01. **(D).** Example flow cytometry plots of CD45RA and CD27 co-expression on Vδ2^neg^ cells in control, CD and CDI donors. **(E).** Flow cytometric analysis of CD103 expression on Vγ4^+^ colonic lymphocytes from paired uninflamed and inflamed tissue sampled at the same endoscopy (n=5). Open circles – CD donor; filled circles – UC donors. Ratio paired *t*-test, **p*<0.05. **(F).** Example flow cytometry plot of of CD103 expression on Vγ4^+^ cells from paired uninflamed and inflamed tissue sampled at the same endoscopy. **(G).** Flow cytometric analysis of IL17A expression by colonic Vγ2/3/4^+^, Vγ2/3/4^neg^, Vδ2^+^ γδ T cells and CD4^+^ and CD8^+^ αβ T cells (y axis) following 4h of P+I stimulation of samples derived from control (n=6-9), CD (n=7-8), CDI (n=3-4), UC (n=1-4), UCI (n=2-4) donors (x axis). Kruskal-Wallis test with Dunn’s correction against control donors was used for analysis, **p*<0.05. **(H,I).** Flow cytometric analysis of (H) TNFα and (I) IFNγ expression on colonic Vγ2/3/4^neg^, Vδ2^+^ γδ T cells and CD4^+^ and CD8^+^ αβ T cells (y axis) following 4h of P+I stimulation of samples derived from control (n=7-11), CD (n=10-11), CDI (n=4-7), UC (n=2-6), UCI (n=2-4) donors. Kruskal-Wallis test with Dunn’s correction against control donors was used for analysis, **p*<0.05; ***p*<0.01.

**Figure S5. The cytokine milieu can affect the phenotype of intestinal Vγ4^+^ cells. (A).** Gene expression analysis of *IL12*, *IL23*, *IL18* in whole gut tissue from control (n=20), IBD (n=19), IBDI (n=21) donors and *TNF* in whole gut tissue from control (n=19), IBD (n=19), IBDI (n=19) donors. Open circles - CD donors; filled circles – UC donors. Data normalized to housekeeping gene *RPS9*. Kruskal-Wallis test with Dunn's correction against control donors was used for analysis, *p**<0.05; *p***<0.01. **(B).** Normalized gene count of *IL18R1*, *IL12RB1, IL12RB2, IL23R, IL1R1, ILR2, TNFRSF1A* and *TNFRSF1B* in Vγ4^+^ cells from control donors (n=4). Data derived from RNA sequencing experiment in Table S3.

**Figure S6. A genetic influence on the Vγ4-BTNL axis. (A).** BTNL8*3 genotyping by standard PCR method. Schematic of genomic organisation and primer binding sites shown above. Representative agarose gel pictures of PCRs shown below. Predicted band size for *BNTL8*3* – 1602 bp; for *BTNL8* – 1310 bp. **(B).** Alignment of reference *BTNL3*, *BTNL8* and predicted *BTNL8*3* sequences to *BTNL8*3* sequences obtained from copy number variation (CNV) homozygous donors (n=6). Gray – non-homologous recombination (NAHR) region. Cyan – sequences identical to *BTNL8* reference. Green – sequences identical to *BTNL3* reference. Bold underlined – potential recombination motif. Fuchsia – SNP candidate rs72494581 for linkage disequilibrium (LD) with the *BTNL8*3* CNV. Numbers – chromosomal position (reference assembly GRCh38.p13). **(C).** TaqMan assay of SNP rs72494581 on the donors from A. **(D).** Flow cytometric analysis of FLAG-tagged BTNL3 (L3) (second row), BTNL8 (L8) (third row) and BTNL8*3 (L8*3) fusion (bottom row) protein expression in the presence of the indicated untagged proteins (labels) in transfected 293T cells. Expression 48h post-transfection. GFP – transfection efficiency reporter. **(E).** Flow cytometric analysis of % Vγ4^+^ cells of Vδ2^neg^ colonic γδ T cells from control (n=23), CD (n=14), CDI (n=5), UC (n=6), UCI (n=8) biopsies. Kruskal-Wallis test with Dunn’s correction against control donors was used for the analysis, **p*<0.05.

Table S1-4 – please see separately uploaded excel files

Table S1. Study participant data

Table S2. All significant DEGs Vg234 CD103 vs neg

Table S3. Top 50 DEGs Vg4 vs Vg589

Table S4. Outcome data

Data S1 – please see separately uploaded excel file
